# Deacidification of the Endolysosomal System by the Vesicular Proton Pump V-ATPase Inhibitor Bafilomycin A1 Affects EGF Receptor Endocytosis Differently in Endometrial MSC and HeLa Cells

**DOI:** 10.3390/ijms262010226

**Published:** 2025-10-21

**Authors:** Anna V. Salova, Tatiana N. Belyaeva, Ilia K. Litvinov, Marianna V. Kharchenko, Elena S. Kornilova

**Affiliations:** 1 Institute of Cytology, Russian Academy of Sciences, Tikhoretsky Av. 4, 194064 Saint-Petersburg, Russia; tatbelyaeva@gmail.com (T.N.B.); lik314@mail.ru (I.K.L.); kharchenko_m@incras.ru (M.V.K.); 2 Higher School of Biomedical Systems and Technologies, Peter the Great St. Petersburg Polytechnic University, Khlopina Str. 11, 195251 Saint-Petersburg, Russia

**Keywords:** mesenchymal stem/stromal cells, tumor-derived cells, EGF receptor, endocytosis, endocytic compartments, Bafilomycin A1, confocal microscopy

## Abstract

It is well-known that EGF binding to EGFR stimulates signal transduction and endocytosis, with the latter leading to lysosomal degradation of EGFR. However, the majority of data on the regulation of endocytosis have been obtained in tumor-derived cells. Here, we perform a comprehensive analysis of the role of endolysosome acidification in the regulation of endocytic pathway in tumor cells and in endometrial MSCs as a model of proliferating, undifferentiated, non-immortalized cells. Using QD-labeled EGF, the dynamics of co-localization of EGF-receptor complexes with endocytic markers in the control and upon inhibition of V-ATPase by Bafilomycin A1 (BafA1) were studied using confocal microscopy. Image analysis showed that in HeLa and A549 cells, BafA1 significantly slowed down EGFR entry into and exit from EEA1-positive early endosomes without disrupting passage through Rab7, CD63, and Lamp1 compartments, but rather shifting it to later times. In enMSCs, only a portion of EGF-containing endosomes entered the degradation pathway, and lysosomal delivery was significantly delayed. Unlike HeLa, in enMSC early endosomes, BafA1 increased the association of EGF-QDs with EEA1, suggesting a lower pH level, which is suboptimal for EEA1-dependent fusions. It is concluded that, unlike HeLa, enMSCs form a population of pH-independent endosomes containing activated EGFR for a long time.

## 1. Introduction

Since the 1980s, it has been established that the binding of the epidermal growth factor receptor (EGFR/c-ErbB1) with its ligands initiates signaling from the intrinsic tyrosine kinase receptor domain at the plasma membrane, as well as internalization of ligand–receptor complexes into endosomes. The role of endosomal EGFR in signal maintenance/generation is now widely accepted [1,2,3,4].

It should be noted that almost all canonical concepts related to EGFR endocytic pathway regulation are based on studies of immortalized cells of tumor origin, such as HeLa, A431, A549, etc., expressing a high level of the receptor (according to estimates, more than 100,000–200,000 molecules per cell, as opposed to less than several thousand in normal cells), and, generally, no fundamental differences have been revealed. The idea of the universality of EGFR endocytosis regulation and the positive relationship between EGFR overexpression and malignant cell transformation have become widespread. However, the question of whether the regulation of EGFR signaling and endocytosis differs in transformed and normal cells remains unresolved, primarily due to the challenges of identifying an appropriate model of “normal” cells and the difficulties in working with cells expressing low levels of the receptor. Primary cells from different tissues of healthy donor should be such a model; however, they are finally differentiated and adapted for specific tissue function that differ them from immortalized partially dedifferentiated tumor-derived cells with elevated proliferative potential. Nonetheless, undifferentiated mesenchymal stem/stromal cells (MSCs) in the proliferative phase can be considered a model of normal cells, since they are non-immortalized, responsive to extracellular stimuli, undergo senescence rather than transformation, and do not form xenografts [5,6,7]. As we have previously demonstrated, the EGFR is the only member of the four c-ErbB family receptors that is detected at the protein level in MSCs of various tissue origins, including endometrial-derived MSCs. These cells respond to EGF by stimulation of proliferation [8,9]. However, detailed studies of the organization of the endolysosomal apparatus and the endocytosis of ligand–receptor complexes in MSCs, as far as we know, are absent.

The prevailing hypothesis concerning the main mechanism of EGFR internalization subsequent to ligand binding and dimerization is clathrin-mediated endocytosis. Dimerized ligand–receptor complexes are sorted from the plasma membrane in endocytic vesicles that recruit small GTPase Rab5, and then are delivered to early Rab5- and EEA1-positive endosomes by multiple fusions. From here, depending on the ligand, the receptor can be recycled back to the cell surface via recycling endosomes (in the case of TGF-*α*, which dissociates in a mildly acidic environment of early endosomes at pH 6.0–6.2) or transported to late endosomes, which are often considered to be multivesicular bodies (MVBs), and further to lysosomes for degradation (in the case of EGF, which dissociates from EGFR at pH lower than 5.0) [10,11,12,13]. Consequently, Rab7 and tetraspanin CD63 are applied as markers of MVBs. Also, CD63, along with Lamp1, is considered to be a marker of lysosomes. Importantly, activated EGFR may be involved in signaling only when its tyrosine kinase domain is exposed into the cytoplasm. It loses its ability in cases of dissociation from the ligand or after transition into the intraluminal vesicles of MVBs, a process designated as maturation. Due to the highly dynamic nature of vesicular transport and the asynchronous progression along the endocytic pathway the relationships between late compartments of the degradative pathway and the mechanisms regulating them are still far from fully understood.

One of the earliest and most common properties of the endolysosomal apparatus was the finding that endosomes and lysosomes are acidified [14,15,16,17]. It is currently believed that the pH level ranges from 6.8 to 6.0 in early endosomes, from approximately 5.5 to 5.0 in late endosomes, and from 5.0 to 4.3 in lysosomes [18,19,20]. The vesicular proton pump V-ATPase was shown to be responsible for endosomal acidification. Maintaining the proper pH within various endocytic compartments is crucial for regulating a series of critical processes, including vesicular fusions/fissions, cargo sorting and maturation processing along the endocytic pathway, as well as the activation of lysosomal acidic hydrolases and mTORC1 signaling [21,22,23,24,25,26].

V-ATPase consists of two multisubunit sectors, *V*_0_ and *V*_1_. *V*_0_ sector is integrated into the endosomal membrane and transports protons into the lumen of the compartment as a result of ATP hydrolysis by subunits of *V*_1_ sector when it is associated with *V*_0_ form. Subunit *a* of *V*_0_ has four isoforms and stabilizes the association of *V*_0_ with *V*_1_. It is also assumed to function as a binding site for Rab5 on endocytic vesicles and Rab5, Arf1, and tether protein EEA1 on early endosomes. Furthermore, it is believed to serve as an intraluminal pH sensor for Ca^2+^ efflux from the endosomal lumen within the pH range characteristic of early endosomes. These events lead to calmodulin-dependent SNARE complex activation followed by fusions of endocytic vesicles with early EEA1-positive endosomes. Ca^2+^ also play a pivotal role in pH-dependent fusions in the late stages of endocytosis. These interactions indicate that the V-ATPase is positioned as a hub in a network of molecular events regulating endocytosis. Extracellular stimuli such as glucose level, amino acid starvation, or EGF may regulate V-ATPase by stimulating the association of *V*_0_ with *V*_1_ [27,28,29,30,31,32,33].

In this study, we used macrolide antibiotic Bafilomycin A1 (BafA1), which has been shown to be a specific inhibitor of V-ATPase [34,35,36,37]. To the best of our knowledge, detailed studies examining the effect of acidification suppression by BafA1 on the dynamics of EGF-receptor endocytosis in immortalized tumor cell lines in comparison with normal undifferentiated cells are absent. In the present study, the endocytosis was stimulated by EGF bound to CdSe/ZnS–PEG quantum dots (QDs) via a biotin–streptavidin linker (hereinafter referred to as EGF-QDs). Previously, we showed that such a complex behaves similarly to the native ligand [38]. The immunofluorescence analysis of immortalized tumor-derived cell lines HeLa and A549 and three non-immortalized human endometrial-derived MSC lines (enMSCs) using antibodies against endolysosomal markers was employed to quantitatively characterize the dynamics of endocytosis in control and after the addition of BafA1 using two different protocols.

In the present paper, it is shown that, in enMSCs compared to HeLa, a significantly lower portion of internalized EGF-receptor complexes entered the canonical endolysosomal pathway, but the order of passing through endosomal compartments was similar. However, the key stages that differ significantly in the two cell types were identified as (i) entry into early endosomes and (ii) the transition from the late CD63 compartment to the Lamp1 lysosome. Pretreatment with BafA1 failed to prevent the internalization of ligand–receptor complexes and their sequential passage through early EEA1-positive endosomes, Rab7- and CD63-positive compartments, and delivery to Lamp1-lysosomes. However, the dynamics of both sorting into these compartments and segregation from them was slowed down in both cell types. The EEA1-dependent fusion of early endosomes and exit from them, and sorting into CD63-positive vesicles, turned out to be the most sensitive to BafA1 treatment. Our findings may indicate the presence of a lower pH in early endosomes of enMSCs, which is suboptimal for fusions of newly formed receptor-containing endocytic vesicles and early EEA1-positive endosomes.

## 2. Results

### 2.1. Dynamics of Endolysosome Deacidification During Pretreatment of Cells with BafA1

**Figure 1:**
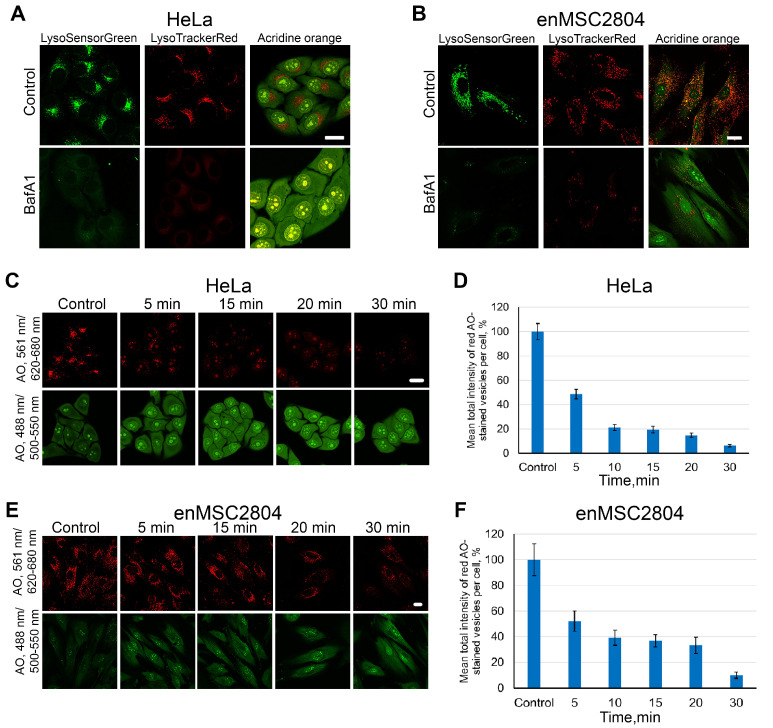
Deacidification of endolysosomes by BafA1 in HeLa and enMSC2804 cells. (**A**,**B**) Confocal images of HeLa (**A**) and enMSC2804 (**B**) cells in control conditions and after pretreatment with BafA1 (100 nM, 30 min), stained with various vital dyes: LysoSensor Green (1 μM, 30 min), LysoTracker Red (50 nM, 30 min), Acridine Orange (AO, 2.5 μg/mL, 1 min). (**C**,**E**) Confocal images of HeLa (**C**) and enMSC2804 (**E**) cells in the control condition and after pretreatment with BafA1 (100 nM) for different periods of time (5, 15, 20, and 30 min). Cells were stained with AO (2.5 μg/mL, 1 min) before confocal imaging. AO fluorescence was excited using 488 nm and 561 nm lasers and detected within two spectral regions: 500–550 nm (green channel, the monomeric form of the dye in the cytoplasm and nucleus under non-acidic conditions) and 620–680 nm (red channel, the stack form of AO in endolysosomes). The brightness and contrast were only adjusted for presentation. Each image is a representative of at least three independent experiments. Scale bars: 10 μm. (**D**,**F**) Mean total intensity of red AO-stained granules per cell. At least 50 cells in five different fields were analyzed for every experimental point. The results are presented as the mean ± SEM.

When V-ATPase activity is inhibited by BafA1, a new ionic balance in the lumen of the acidified vesicles is not established instantly. Therefore, the prevailing protocol for this inhibitor application is to add it to the cells 20–30 min prior to stimulating endocytosis and maintain its presence in the incubation medium throughout the experiment [35,39]. In order to qualitatively evaluate the dynamics of BafA1 action, three known vital fluorescent dyes that accumulate in acidified compartments were utilized: LysoSensor Green, LysoTracker Red, and Acridine Orange (AO). All three probes are known to diffuse freely across intact plasma membranes of living cells into the cytoplasm and subsequently into acidified compartments, primarily lysosomes and possibly late endosomes. At these compartments, the probes become protonated and are unable to diffuse back across the organelle membrane, resulting in a vesicle accumulation. AO exists as monomers at low concentrations in the cytoplasm, and exhibits green fluorescence. However, when accumulated in acidic compartments, the protonated form of AO forms dimers and oligomers that fluoresce in the red channel [40,41]. As illustrated in Figure 1A (upper panel), in control HeLa cells, LysoSensor and LysoTracker exhibit strong fluorescent signals exclusively within vesicular structures concentrated in the perinuclear region. AO reveals similar perinuclear vesicular clusters that exhibit a red fluorescence in the range of 620–680 nm, with the cytoplasm and nucleus also exhibiting a weak green staining at 500–550 nm. The staining of enMSCs with these three dyes (Figure 1B, upper panel) demonstrates a similar fluorescence character as observed in HeLa, yet it reveals that the acidified compartment is represented by numerous vesicles distributed almost throughout the cell volume, with a lower degree of clustering in the perinuclear region compared to HeLa.

Treatment of HeLa cells with BafA1 for 30 min, followed by the addition of dyes, reveals a near-total absence of vesicular fluorescence in all three cases, with AO still detectable in the cytoplasm and nucleus (Figure 1A, lower panels). Vesicular staining is also absent in enMSCs (Figure 1B, lower panels). However, the dynamics of the AO fluorescence decrease in vesicular structures of enMSCs (Figure 1E,F) were comparatively slower than that of HeLa (Figure 1C,D), and, in the initial 10 min of exposure, the fluorescence decreased by only 60% rather than by 80% in HeLa.

### 2.2. EGF Receptor Endocytosis Under BafA1 Pretreatment in Tumor-Derived and MSC Cells

**Figure 2:**
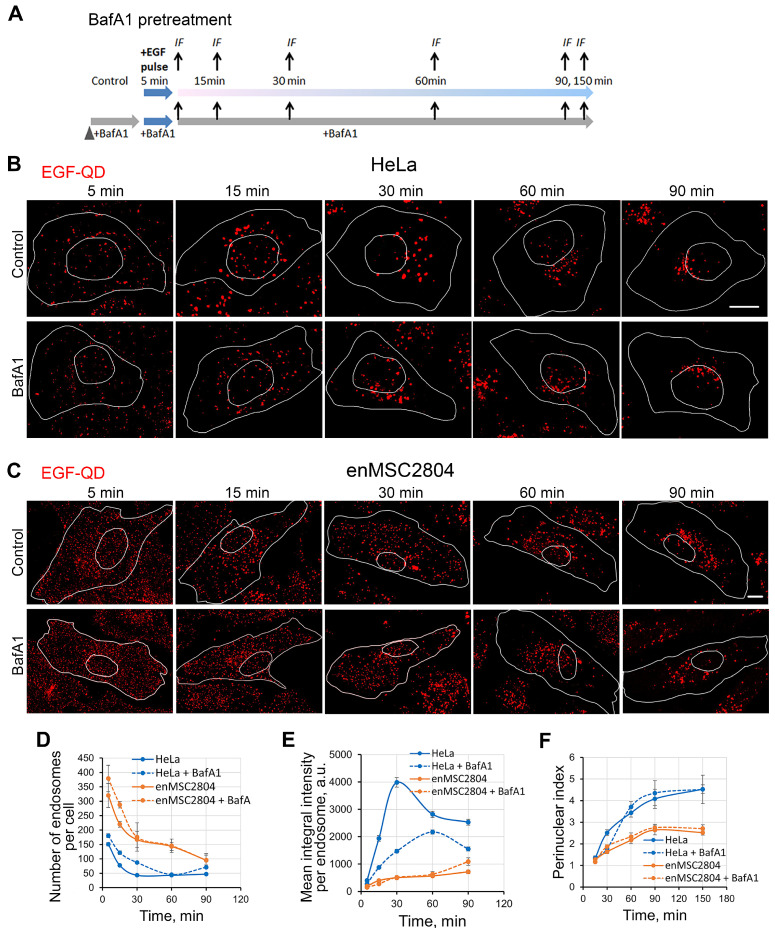
Time-dependent comparison of EGF-QDs distribution during endocytosis in HeLa and enMSC2804 cells in control conditions and after BafA1 pretreatment. (**A**) The experimental design for the BafA1 pretreatment is shown. In both control cells and cells with BafA1 pretreatment (100 nM, 30 min) endocytosis was stimulated using the pulse-chase (5 min) protocol with EGF-QDs. For immunofluorescence, samples were fixed at a specified times from the start of stimulation. BafA1 was present throughout the entire experiment. (**B**,**C**) Confocal images of HeLa (**B**) and enMSC2804 (**C**) cells that were incubated with EGF-QDs (red signal) according to the experimental design described in (**A**). Each image is a maximum intensity projection of a Z-stack onto a single image, representative of the corresponding time point in at least three independent experiments. The brightness and contrast were only adjusted for presentation. The white lines in the images delineate the boundaries of the cells and nuclei according to differential interference contrast. Scale bars: 10 μm. (**D**) The number of endosomes per cell, (**E**) the mean integral intensity of QD fluorescence per endosome, and (**F**) the perinuclear index (the ratio of the area of the whole cell to the area occupied by the bulk of endosomes) were calculated at each time point using Fiji software 1.52v for a series of experiments with representative images in (**B**,**C**). All measurements were performed in three independent experiments, with a total of ∼150 cells per time point. The results are presented as the mean ± SEM.

We have compared the dynamics of endocytosis of EGF-receptor complexes after stimulation with EGF-QDs in HeLa and enMSC2804 cells under control conditions and after BafA1 pretreatment according to the experimental design shown in Figure 2A. Briefly, the EGF-QD complex, prepared as described in Section 4 was added to the cells for 5 min at 37 °C. Since the coated pit turns into an endocytic vesicle within 2–5 min [10], the majority of ligand-bound receptors are internalized during the 5 min pulse, thereby stimulating a synchronous wave of endocytosis [38]. Unbound ligands were washed away, and incubation was continued for 90–150 min. According to the pretreatment protocol (Figure 2A), BafA1 was added 30 min prior to the stimulation of endocytosis with the ligand and then was continuously present in the incubation medium, leading to a maximal deacidification of the intracellular acidic compartments without significant side effects.

Under our conditions, the formation of functional ligand–receptor complexes was observed, since almost all EGF-QDs co-localized with EGFR with a high Manders’ coefficient of approximately 0.8 in both HeLa cells (Supplementary Figure S1A,B) and enMSCs (Supplementary Figure S1C,D). EGF-QDs remained in a complex with the receptor throughout the entire experiment, thus maintaining its activity (Supplementary Figure S1E,F). It is essential to emphasize that QDs serve as a measure of the cargo delivery into lysosomes and its subsequent accumulation there, without being subjected to degradation. The employment of QDs enables us to separate the processes of cargo entry into lysosomes and its degradation by lysosomal enzymes.

The mean intensity of EGF-QD-containing endosomes in HeLa cells increased dramatically by 30 min, whereas in enMSCs it raised slightly only in the first 15 min after endocytosis stimulation; however, it was by seven- to eight-fold lower than the peak intensity value in HeLa cells (Figure 2D,E). These data indicate that the efficacy of endosome fusions and cargo concentration in HeLa cells is maximal at the early stage of endocytosis and is significantly higher than that observed in enMSCs. A comparison of the corresponding cell images (Figure 2B,C, control) reveals that, whereas in HeLa cells the vast majority of endosomes are enlarged already after 15 min, in enMSCs, the predominant population of EGF-QD-containing endosomes consists of small dim vesicles. Furthermore, the differences in the decrease in the vesicle number and the increase in their intensity in HeLa cells are consistent with the hypotheses concerning the formation of multivesicular endosomes with dense packing of intraluminal vesicles, to which EGF-receptor complexes are translocated. In enMSCs, only a few large vesicles are visible in the perinuclear region of the cells after 30 min of endocytosis.

A distinguishing feature of the endocytic pathway of the receptor is its vectorality, provided by dynein-dependent transport of endosomes along microtubules to the perinuclear region, where lysosomes are mainly localized [42]. However, endosome clustering was significantly higher in HeLa cells than in enMSCs (Figure 2F), despite the presence of a well-formed radial microtubule system in both cell lines.

The endolysosome deacidification induced by BafA1 did not prevent the internalization of EGF-receptor complexes, although it slightly increased the number of endosomes at the early stages in both cell lines (Figure 2B–E, BafA1). Furthermore, the mean endosome intensity in HeLa cells increased less than in the control conditions and reached its maximum after 60 min, i.e., at later times. It can be assumed that, in HeLa cells, deacidification reduced the efficiency of the both early endocytic fusions and the subsequent formation of internal vesicles of MVB, although these processes do still occur. In enMSCs under BafA1 treatment, only a slight increase in endosome number was observed at an early stage, while the intensity remained virtually unchanged. This finding can be interpreted as a very low efficiency of early fusions that does not lead to the formation of significant amounts of MVB with concentrated cargo. Also, deacidification does not significantly affect the dynamics of endosome clustering in the perinuclear region in both HeLa cells and enMSCs (Figure 2F).

### 2.3. Effect of BafA1 Pretreatment on the Dynamics of the Main Stages of EGF Receptor Endocytosis Stimulated by EGF-QDs in Tumor and Mesenchymal Stromal Cells

**Figure 3:**
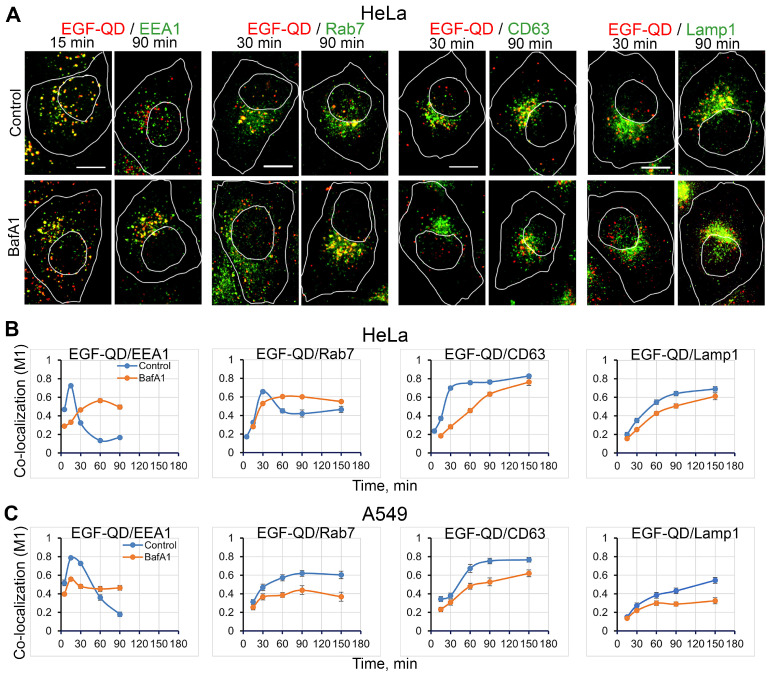
Co-localization dynamics of EGF-QDs with specific endocytic markers in cancer cell lines in control conditions and after BafA1 pretreatment. (**A**) Confocal images of HeLa cells that were incubated with EGF-QDs (red) according to the experimental design described in Figure 2A. The cells were then fixed and immunostained with an anti-EEA1, anti-Rab7, anti-CD63, and anti-Lamp1 antibodies (green). Each image is a maximum intensity projection of a Z-stack onto a single image, representative of the corresponding time point in at least three independent experiments. The brightness and contrast were only adjusted for presentation. The white lines in the images delineate the boundaries of the cells and nuclei according to differential interference contrast. Scale bars: 10 μm. (**B**,**C**) Manders’ coefficients (M1) of co-localization between EGF-QDs and specific endocytic markers in HeLa cells (**B**), obtained from a series of experiments with representative images of cells shown in (**A**), and A549 cells (**C**); images not provided. All measurements were performed in three independent experiments, with a total of ∼130 cells per time point. The results are presented as the mean ± SEM.

**Figure 4:**
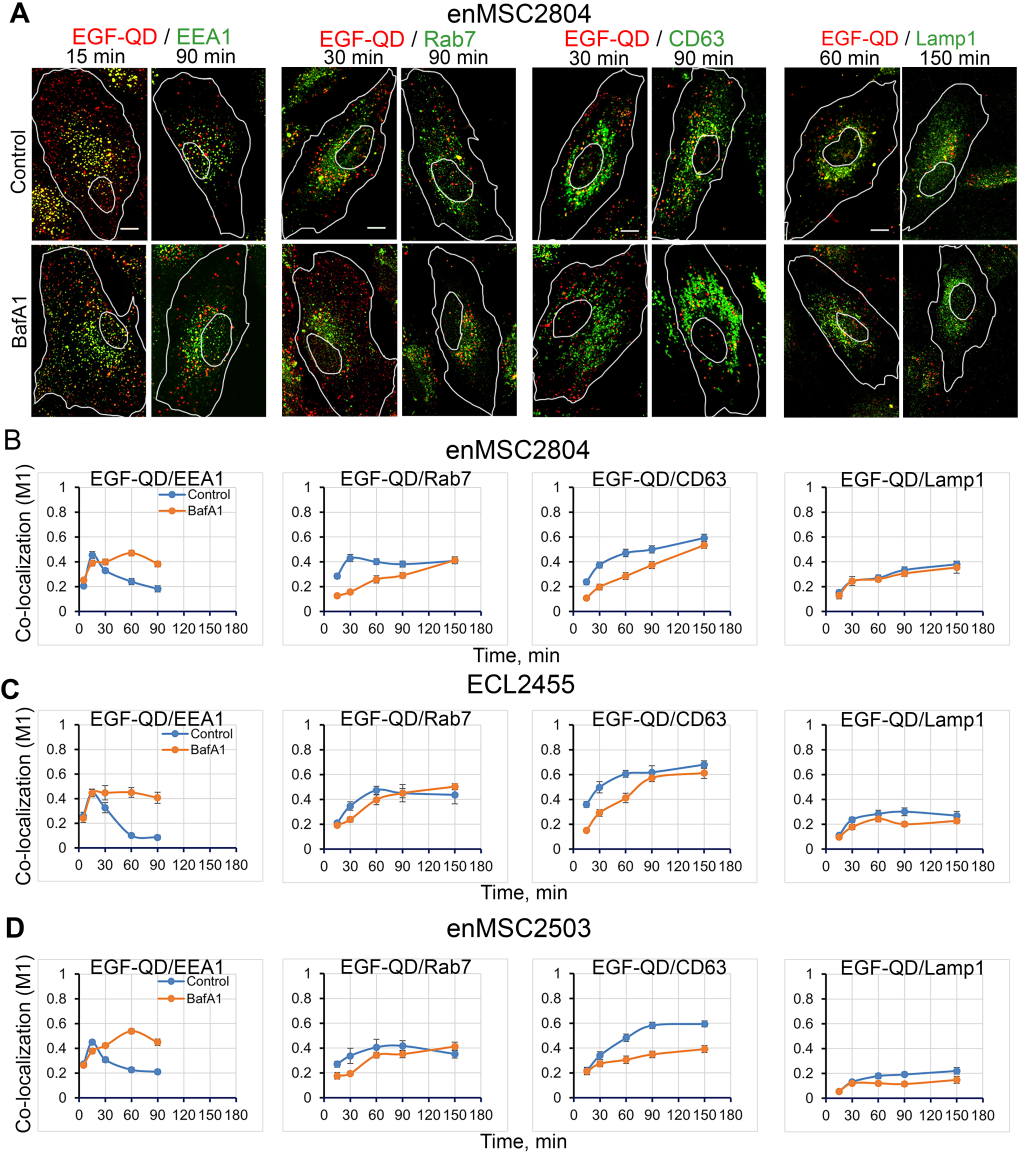
Co-localization dynamics of EGF-QDs with specific endocytic markers in mesenchymal cell lines in control conditions and after BafA1 pretreatment. (**A**) Confocal images of enMSC2804 cells that were incubated with EGF-QDs (red) according to the experimental design described in Figure 2A. The cells were then fixed and immunostained with anti-EEA1, anti-Rab7, anti-CD63, and anti-Lamp1 antibodies (green). Each image is a maximum intensity projection of a Z-stack onto a single image, representative of the corresponding time point in at least three independent experiments. The brightness and contrast were only adjusted for presentation. The white lines in the images delineate the boundaries of the cells and nuclei according to differential interference contrast. Scale bars: 10 μm. (**B**–**D**) Manders’ coefficients (M1) of co-localization between EGF-QDs and specific endocytic markers in enMSC2804 cells (**B**), obtained from a series of experiments with representative images of cells shown in (**A**), ECL2455 cells (**C**), images not provided, and enMSC2503 cells (**D**), images not provided. All measurements were performed in three independent experiments, with a total of ∼100 cells per time point. The results are presented as the mean ± SEM.

Next, a more detailed study was conducted to ascertain the impact of BafA1 pretreatment on the sequential stages of the endocytic pathway. Using immunofluorescence analysis, the dynamics of EGF-QD co-localization with antibodies against markers of early endosomes (Rab5, EEA1), multivesicular/late endosomes (Rab7, CD63), and lysosomes (Lamp1) were investigated in two typical tumor cell lines (HeLa, A549) and three enMSC lines (enMSC2804, ECL2455, enMSC2503). The stages of endocytosis were characterized by calculating the Manders’ co-localization coefficient (M1) of EGF-QD-containing structures with these markers (Figure 3 and Figure 4).

The co-localization of EGF-QDs with EEA1 in control HeLa cells was maximal 5–15 min after endocytosis stimulation (M1 ≈ 0.5–0.7) and decreased to background levels after 30 min (Figure 3B) and later. By 90 min, EGF-QDs and EEA1 were found in completely different structures (Figure 3A). After 30 min, EGF-QDs and Rab7 showed maximal co-localization (Figure 3A,B), which is consistent with the dynamics of the release of EGF-receptor endosomes from the EEA1-positive early endosomes already in the form of multivesicular structures. Interestingly, the association of EGF-QDs with CD63 also reached a maximum at 30 min (Figure 3B), but while the association with Rab7 passed through a maximum at 30 min and then decreased, the co-localization with CD63 reached a plateau (M1 ≈ 0.8), which may indicate the existence of an endosomal population carrying only CD63. However, the corresponding cell images (Figure 3A, control, EGF-QD/Rab7 and EGF-QD/CD63) demonstrated that Rab7 and CD63-positive structures were localized in the perinuclear region, while EGF-QD/Rab7- and EGF-QD/CD63-containing structures were located at the edge of this cluster at 30 min and then moved inside it, where they were retained until late stages. It is noteworthy that, even in the early stage, no co-localization of EGF-QDs with these two markers was detected in peripheral endosomes in HeLa cells. Then, in accordance with classical concepts, after 60 min, a significant fraction of EGF-QD structures were found to co-localize together with the lysosomal marker protein Lamp1 (Figure 3A,B), thereby reflecting the dynamics of protein cargo delivery to lysosomes. A comparison of the co-localization degree of EGF-QDs/CD63 with EGF-QDs/Lamp1 suggests that, at this late stage of endocytosis, not all EGF-receptor complexes are co-localized with Lamp1, but some of them remain co-localized only with CD63.

Pretreatment of HeLa cells with BafA1 (Figure 3A,B, BafA1) demonstrated that the most pH-dependent stage of EGF-receptor complex endocytosis was the fusion of endocytic vesicles with EEA1-positive early endosomes. However, in the absence of acidification, the EGF-QD association with EEA1 was strongly slowed down, though not suppressed, shifting the fusion/enlargement of EEA1-positive endosomes for a longer period. Interestingly, if in the control Rab7 was associated with large EGF-QD-containing vesicles located actually at the edge of the perinuclear cluster of Rab7-positive structures after 30 min, then, in the presence of BafA1, this marker was associated with smaller, more peripheral EGF-QD-containing endosomes. Sorting into the CD63-positive compartment was also significantly delayed, while the cargo delivery to lysosomes was slowed down less, although numerous studies, including our own, have demonstrated that BafA1 completely blocks EGFR degradation [43,44].

A similar experiment on co-localization of EGF-QDs with markers of the endocytic pathway in the control and under deacidification conditions on A549 tumor cells, which express a high number of EGFR, demonstrated the variability of endocytosis dynamics in tumor models (Figure 3C, confocal images not provided). In general, the main characteristics of endocytosis of EGF-receptor complexes are similar in tumorigenic cell lines HeLa and A549 (Figure S2 and Figure 3C), and the most potent effect caused by the inhibition of V-ATPase activity is the significant suppression of fusions of EGF-QD-containing endocytic vesicles with early EEA1-positive endosomes and subsequent homotypic EEA1-dependent fusions at the initial step of the endocytic pathway. Additionally, the exit of EGFR from hybrid early endosomes was slowed.

Next, we analyzed the effect of BafA1 pretreatment on EGF-QD endocytosis in three endometrial MSC lines (Figure 4), two of which were derived from desquamated endometrium (enMSC2804 and enMSC2503) and the third from biopsy material (ECL2455). All three lines (Figure 4B–D) exhibit a comparable sequential association of EGF-QDs with EEA1 (15 min, maximal M1 ≈ 0.5), then with Rab7 (at 30 min M1 ≈ 0.48 and remains at this level) and CD63 (starting at 30 min with slow growth of M1 up to approximately 0.6 by 150 min); however, notable discrepancies were observed compared to tumor cells (Figure 3). Firstly, the proportion of EGF-QDs maximally associated with markers in enMSCs was noticeably lower than that in HeLa. This indicates that, in enMSCs, not all newly formed endocytic vesicles enter the canonical EEA1 cycle. Those that do form hybrid EEA1-positive early endosomes exit them and form vesicles carrying Rab7 and/or CD63 more slowly, both in the control condition and upon BafA1 pretreatment. However, the highest degree of co-localization of EGF-QDs in both HeLa and enMSCs was observed with CD63, rather than with Rab7 and Lamp1, and this stage was significantly dependent on the endosome deacidification by BafA1. Furthermore, in contrast to tumor cells (Figure 3), all enMSC lines did not exhibit EGF-QD accumulation within Lamp1-positive lysosomes during at least 150 min of the experiment, which was also confirmed by the independence of co-localization of the cargo and this marker when V-ATPase activity was inhibited by BafA1.

It should be emphasized that the difference in the effect of BafA1 on the behavior of EGF-QDs in HeLa and enMSCs was most pronounced at the stage of interaction of EGF-QDs with early EEA1-positive endosomes. In contrast to HeLa cells, in all three enMSC lines BafA1 pretreatment prolonged EGF-QD association with EEA1 instead of suppressing it (Figure 4B–D, EGF-QD/EEA1 graphs).

### 2.4. The Effect of Suppression of V-ATPase Activity on the Sorting Process of EGF-Receptor Complexes Within Endocytic Pathway Compartments

The data presented above indicate that the deacidification of the endolysosomal apparatus did not completely block endocytosis and did not affect the order of passage through compartments, but significantly slowed down the entry of cargo into compartments, as well as its exit from them. This can affect the sorting process, resulting in the localization of EGF-QDs in structures that simultaneously carry all markers of different stages of endocytosis.

To test this assumption, a triple co-localization analysis was conducted using a two-by-two comparison. For this purpose, we identified the co-localization of vesicles containing EGF-QDs, first with one marker, then with the other, thereby obtaining artificially colored red and green masks of co-localized structures, respectively. Subsequently, the overlapping areas of these masks were identified, and they were assigned a white color, which reflected the structures carrying all three signals. Importantly, EGF-QD-receptor complexes were presented in all the areas identified. For comparison, the total area of all EGF-QD-containing structures in the cell for each time point was accounted for 100%, and the resulting areas of double and triple overlaps were normalized to this value. The results are presented as histograms, with the fractions obtained from triple co-localization highlighted in white, those from double co-localization for each marker separately highlighted in green and red according to the figure captions, and those containing only EGF-QDs in gray.

To verify the approach, the triple co-localization of EGF-QDs, EEA1, and Lamp1, markers of the most distant initial and final points of the degradative endocytic pathway which do not normally intersect, was assessed in HeLa cells (Supplemental Figure S3). The cell images demonstrate (Supplemental Figure S3A) that, in both the control and the BafA1-pretreated cells, triple co-localization was virtually absent. Concurrently, BafA1 slowed down the association of EGF-QDs with EEA1 from early time points (15 min) and further to late stages, and the delivery of EGF-QDs to Lamp1-positive lysosomes underwent a pronounced decrease. Therefore, the triple co-localization remained at the same low level of random coincidences as in the control, indicating that the sorting process was not significantly disturbed at deacidification conditions. An analysis of the triple co-localization of EGF-QDs with EEA1 and Lamp1 markers in enMSCs was not conducted, since the association of EGF-QDs with lysosomes in the control in this case is at background level.

**Figure 5:**
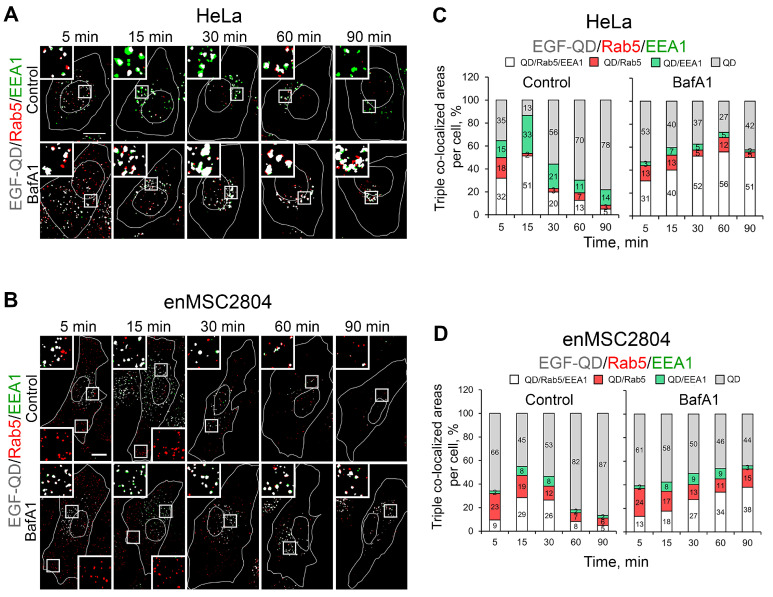
Triple-co-localization analysis of the distribution of EGF-QDs in relation to specific endocytic markers Rab5 and EEA1 in HeLa and enMSC2804 cells in control conditions and after BafA1 pretreatment (100 nM, 30 min). (**A**,**B**) Cell masks in HeLa (**A**) and enMSC2804 (**B**) are shown after applying the triple-co-localization detection method, revealing areas of co-localization of: QDs and Rab5 (red), QDs and EEA1 (green), and the triple QDs, Rab5, and EEA1 (white). The white lines in the images delineate the boundaries of the cells and nuclei according to differential interference contrast. The insets represent enlarged (3×) views of the corresponding boxed regions of the cell. Note that on the images only triple- and double-co-localized areas are presented. Scale bars: 10 μm. (**C**,**D**) Histograms showing the areas occupied by the QD-associated marker proteins Rab5 and EEA1 per cell (triple and double co-localizations), normalized to the total QD-positive area, which include triple and double co-localization and vesicular structures containing only QDs, at each time point and presented as percentages in control conditions and after BafA1 pretreatment in HeLa cells (**C**), obtained from a series of experiments with representative images of cells shown in (**A**), and in enMSC2804 cells (**D**), obtained from a series of experiments with representative images of cells shown in (**B**). All measurements were performed in three independent experiments, with a total of ∼100 cells per time point.

Next, the dynamics of triple co-localization of EGF-QDs with the early endocytosis markers Rab5 and EEA1 in HeLa and enMSC2804 cells were analyzed (Figure 5). After 5 min, a significant overlapping area (18%) of EGF-QDs and Rab5 was identified in control HeLa cells. This area may correspond to transport vesicles that have just been released from the AP-2 coating. However, after 15 min the majority of the overlaps (up to 40% of the area carrying EGF-QD signals) contain all three markers, indicating that they are already located in enlarged early EEA1-positive endosomes (Figure 5A,C). Consequently, the number of structures exhibiting double and triple co-localization decreases to a level of random coincidences, while the proportion of vesicles containing only EGF-QDs increases (gray part of the columns), indicating the release of EGF-receptor complexes from early endosomes. Such dynamics are generally canonical for the initial stage of endocytosis [45,46,47], and correspond to the data obtained from the double co-localization assessment (Figure 3).

Under BafA1 pretreatment, while the total number of vesicles containing EGF-QDs increased (Figure 2D), the structures containing EGF-QDs/Rab5 and EGF-QDs/Rab5/EEA1 after 15 min were less than in the control (Figure 5C, BafA1). However, the proportion of areas with EGF-QDs/Rab5/EEA1 co-localization subsequently increased, mainly due to the fact that it was the triple-co-localized structures that became significantly larger, reaching a maximum of 56% after 60 min, and only then decreased. Concurrently, a greater number of relatively small vesicles containing EGF-QDs and Rab5 are present throughout the experiment, in contrast to the control. These data indicate a slowdown of EGF-QD entry into early EEA1-positive endosomes, as well as of their exit from them.

The same analysis performed on enMSCs showed a similar trend in the dynamics of triple co-localization, both in the control and in the presence of BafA1 (Figure 5B,D), though with two exceptions. Firstly, the proportion of ligand area associated with two early-stage markers was lower in both cases and endosomes were significantly smaller than in HeLa, which coincides with the data shown in Figure 3B and Figure 4B. Secondly, in enMSCs both in the control and in the presence of BafA1, the overlapping area proportion of EGF-QDs and Rab5 only was significantly higher than in HeLa, and was presented by mostly peripheral vesicles throughout the experiment. Upon the BafA1 pretreatment, the proportion of the overlapping area of EGF-QDs and Rab5 signals reached 24% 5 min after stimulation of endocytosis, and remained at a level of about 15% for the entire 90 min of the experiment, whereas in HeLa, such overlaps were below 10%. It should be emphasized that the representative cell images given in Figure 5B reveal that, under deacidification conditions, not only a larger number of ligand-containing early endosomes are present for a long time, but they also increase in size at later stages in comparison to the control. This observation indicates that fusions in enMSCs occurred under conditions that are likely suboptimal for this process, which is positively affected by deacidification.

**Figure 6:**
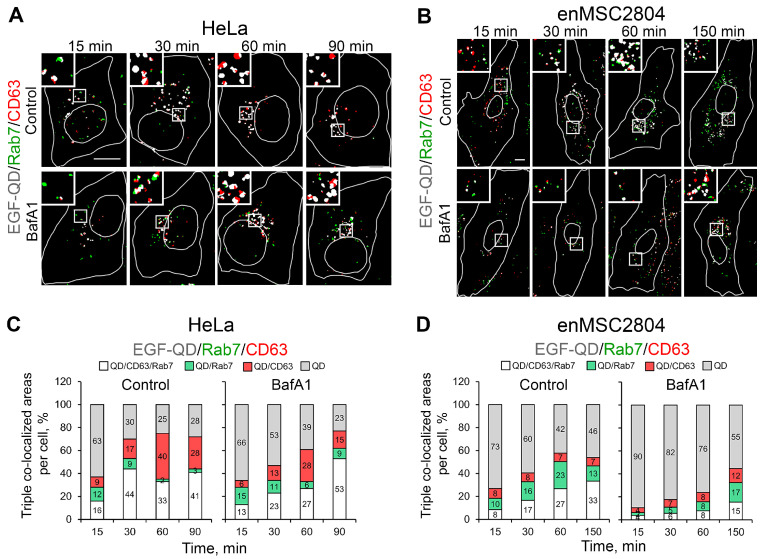
Triple co-localization analysis of the distribution of EGF-QDs in relation to specific endocytic markers Rab7 and CD63 in HeLa and enMSC2804 cells in control conditions and after BafA1 pretreatment (100 nM, 30 min). (**A**,**B**) Cell masks in HeLa (**A**) and enMSC2804 (**B**) are shown after applying the triple co-localization detection method, revealing areas of co-localization of: QDs and Rab7 (green), QDs and CD63 (red), and the triple QDs, Rab7, and CD63 (white). The white lines in the images delineate the boundaries of the cells and nuclei according to differential interference contrast. The insets represent enlarged (3×) views of the corresponding boxed regions of the cell. Note that on the images only triple- and double-co-localized areas are presented. Scale bars: 10 μm. (**C**,**D**) Histograms showing the areas occupied by the QD-associated marker proteins CD63 and Rab7 per cell (triple and double co-localizations), normalized to the total QD-positive area, which includes triple and double co-localization and vesicular structures containing only QDs, at each time point and presented as percentages in control conditions and after BafA1 pretreatment in HeLa cells (**C**), obtained from a series of experiments with representative images of cells shown in (**A**), and in enMSC2804 cells (**D**), obtained from a series of experiments with representative images of cells shown in (**B**). All measurements were performed in three independent experiments, with a total of ∼100 cells per time point.

As demonstrated previously, the degree of association of the EGF-QDs with Rab7 and CD63 began to increase synchronously after the release of EGF-receptor complexes from the EEA1-positive early endosomes after 30 min of endocytosis; however, later on, the co-localization of EGF-QDs and Rab7 decreased, while the co-localization of EGF-QDs and CD63 remained at a high level (M1 about 0.8) (Figure 3B). In order to understand whether there is a separation of the populations of EGF-QD vesicles carrying only CD63 and only Rab7, the dynamics of triple co-localization of the ligand with these two markers in HeLa cells were analyzed. The histograms show (Figure 6C, control) that, after 15 min, EGF-QDs were detected in structures bearing either Rab7 or CD63 alone, as well as those containing all three markers simultaneously. Beginning from 30 min, there was an increase in the proportion of overlapping area with CD63, while the overlapping area with Rab7 decreased. Analysis of cell images (Figure 6A, control) showed that there is not a distinct vesicular population, but rather segregation within the same vesicular structure with the formation of a domain that is positive exclusively for EGF-QDs/CD63 and a domain bearing all three markers. Concurrently, by 90 min, distinct EGF-QDs/CD63 vesicles appeared, exhibiting point-to-point co-localization of EGF-receptor complexes and CD63.

In enMSCs, in contrast to HeLa cells, the proportion of EGF-QD-positive areas that overlap with markers of late stages of endocytosis was expectedly lower, and in the control (Figure 6D), the proportion of EGF-QD/Rab7 area prevails over the EGF-QD/CD63 area. The proportion of areas exhibiting triple co-localization slowly increased with time. The images of masks (Figure 6B, control) reveal that, after 15 min, mainly small vesicles with double co-localization are present, whereas by 30 min and later, CD63 is detected in larger vesicles also containing Rab7. Furthermore, in contrast to HeLa, a population of Rab7-positive endosomes is present up to 150 min of the experiment. Confocal images clearly reveal Rab7 on individual small vesicles, rather than as part of multidomain juxtanuclear-localized structures.

The impact of BafA1 on enMSCs is manifested in the fact that both the number of EGF-containing vesicles carrying the Rab7 and CD63 markers (Figure 6B, BafA1) and the proportion of regions demonstrating triple co-localization (Figure 6D, BafA1) are notably diminished compared to the control. Concurrently, the apparent size of endosomes carrying all three markers is much smaller under deacidification conditions than in the control, which indicates the suppression of late endosome fusions. In addition, the presence of all three populations (EGF-QDs/Rab7, EGF-QDs/CD63, and EGF-QDs/Rab7/CD63) was observed throughout all 150 min of the experiment. It is important to note that, while segregation by markers occurred within the same vesicle in HeLa, the domain organization of the late compartment in enMSCs is not readily apparent.

Confocal images of HeLa cells stained for endocytosis markers show that all markers (Rab7, CD63, Lamp1, and even partly EEA1) are localized in perinuclear structures. In enMSCs, these structures are not so crowded, but nevertheless, markers of the canonical lysosomal degradation pathway also show a tendency toward perinuclear localization. The relationship between vesicles carrying the classical lysosomal marker Lamp1 and structures positive for the tetraspanin CD63, which was initially also considered a lysosomal marker, is a subject of interest [48,49,50].

In the light of the aforementioned findings, a comprehensive analysis was conducted to examine the dynamics of EGF-receptor complexes co-localization with CD63- and Lamp1-positive structures in the control and after BafA1 pretreatment of the cells. For the reasons already mentioned, these also was carried out only on HeLa cells. EGF-QDs were found to be distributed between CD63 domains and domains containing both CD63 and Lamp1 (Supplementary Figure S4). However, over time, there was a notable increase in the area exhibiting triple co-localization. Thus, in control, all structures containing EGF-QDs, also carrying CD63, ultimately were delivered to Lamp1-positive lysosomes. When acidification was suppressed by BafA1, the multidomain nature of the structures containing EGF-QDs was maintained, but in contrast to the control, a number of very small separate vesicles carrying only EGF-QDs and Lamp1 were detected at virtually all stages of the experiment (Supplementary Figure S4).

### 2.5. Effect of Delayed Addition of BafA1 on the Dynamics of EGF-QD Compartmentalization in HeLa Cells and enMSCs

**Figure 7:**
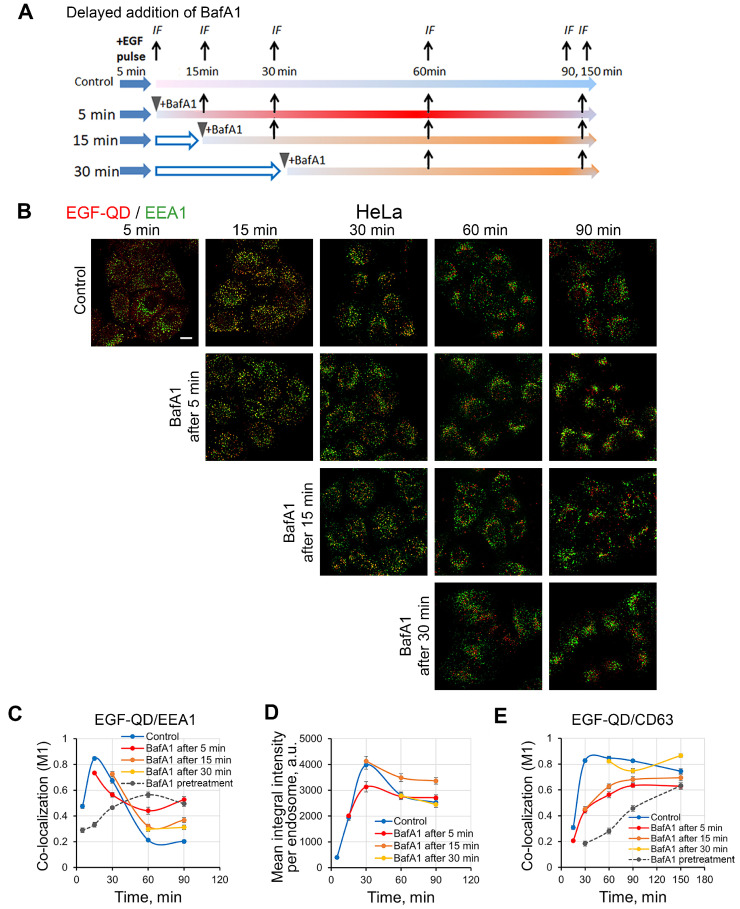
Delayed addition of BafA1 (100 nM) to HeLa cells after endocytosis stimulation with EGF-QDs. (**A**) Experimental design for the delayed addition of BafA1 is shown. Endocytosis was stimulated using the pulse-chase (5 min) protocol for all cases with EGF-QDs. The delayed addition of BafA1 (100 nM) was carried out at the specified times (5, 15, and 30 min, indicated by the arrowhead) from the beginning of endocytosis stimulation. For immunofluorescence, samples were fixed at the specified time from the start of stimulation. (**B**) Confocal images of HeLa cells that were incubated with EGF-QDs (red) according to the experimental design described in (**A**). The cells were then fixed and immunostained with an anti-EEA1 antibody (green). Each image is a maximum intensity projection of a Z-stack onto a single image, representative of the corresponding time point in at least three independent experiments. The brightness and contrast were only adjusted for presentation. Scale bars: 10 μm. (**C**) Manders’ coefficients (M1) of co-localization between EGF-QDs and EEA1 in HeLa cells for a series of experiments with representative images in (**B**). (**D**) The mean integral intensity of QD fluorescence per endosome was calculated at each time point using Fiji software 1.52v for a series of experiments with representative images in (**B**). (**E**) Manders’ coefficients (M1) of co-localization between EGF-QDs and CD63 in HeLa cells for a series of experiments with representative images in Supplementary Figure S3. All measurements were performed in three independent experiments, with a total of ∼130 cells per time point. The results are presented as the mean ± SEM.

Given that the efficiency of EGF-QD association with EEA1 in enMSCs was found to be lower than in HeLa cells, it was proposed that the pH level may differ between the early endosomes of these two cell types and, consequently, EGF-QD endocytic vesicles in enMSCs fail to satisfy the prerequisites for efficacious fusion with EEA1-endosomes. To clarify this point, we stimulated EGF-QD endocytosis in control condition, using 5 min pulse and added BafA1 either immediately or 15 or 30 min after the endocytosis stimulation and then analyzed fixed cells to assess the localization of EGF-QDs and endocytic markers (Figure 7A). According to the data shown in Figure 1, after 5–15 min of BafA1 action, the endosomal pH level is still intermediate between the normal and the maximally deacidified condition.

Using this protocol, we found that, in HeLa cells, the addition of BafA1 immediately after the pulse (Figure 7C, 5 min, red line) resulted in a slightly decreased association of EGF-QDs with EEA1 15 min after the initiation of stimulation (M1 ≈ 0.72, compared to 0.8 in the control), and a delayed release of EGF-QDs from early endosomes was observed. The addition of BafA1 after 15 min and further exhibited minimal impact on the level of co-localization with EEA1. The evaluation of the mean QD signal intensity in endosomes confirmed that the action of the inhibitor, which was added immediately after the pulse (5 min), prevented the accumulation of cargo that accompanies fusions, whereas addition after 15 min maintained the maximum level of intensity, preventing the exit of EGF-QDs from early endosomes without affecting fusions (Figure 7D). Interestingly, the second pH-sensitive step of EGF-receptor complex endocytosis, namely association with the CD63-positive compartment, also was most sensitive to the addition of BafA1 after 5 and 15 min, but not later, while the suppression of EGF-QD entry into this compartment is at an intermediate level between the control and that observed with BafA1 pretreatment (Figure 7E and Figure S5).

**Figure 8:**
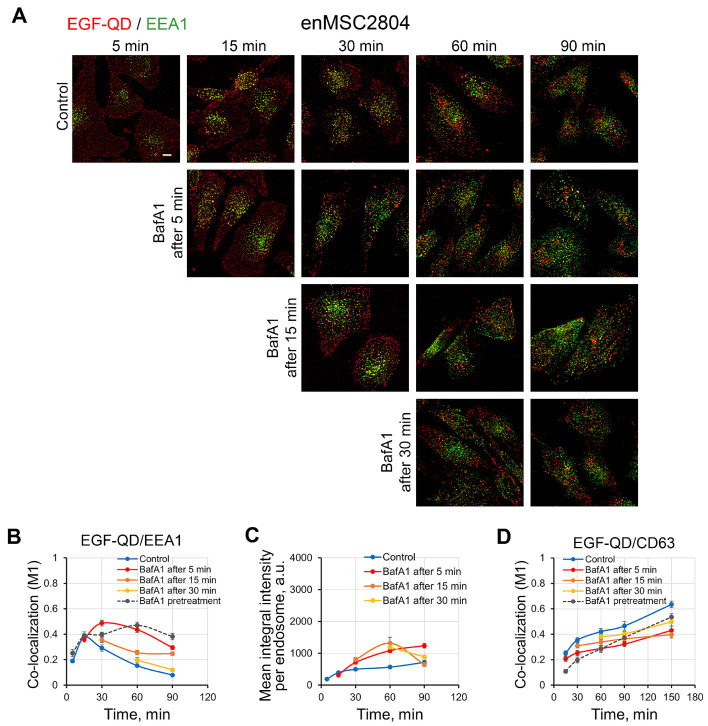
Delayed addition of BafA1 (100 nM) to enMSC2804 cells after endocytosis stimulation with EGF-QDs. (**A**) Confocal images of enMSC2804 cells that were incubated with EGF-QDs (red) according to the experimental design described in Figure 7A. The cells were then fixed and immunostained with an anti-EEA1 antibody (green). Each image is a maximum intensity projection of a Z-stack onto a single image, representative of the corresponding time point in at least three independent experiments. The brightness and contrast were only adjusted for presentation. Scale bars: 10 μm. (**B**) Manders’ coefficients (M1) of co-localization between EGF-QDs and EEA1 in enMSC2804 cells for a series of experiments with representative images in (**A**). (**C**) The mean integral intensity of QD fluorescence per endosome was calculated at each time point using Fiji software 1.52v for a series of experiments with representative images in (**A**). (**D**) Manders’ coefficients (M1) of co-localization between EGF-QDs and CD63 in HeLa cells for a series of experiments with representative images in Supplementary Figure S4. All measurements were performed in three independent experiments, with a total of ∼100 cells per time point. The results are presented as the mean ± SEM.

Similar experiments on enMSC2408 yielded divergent outcomes (Figure 8): addition of BafA1 immediately after a 5 min pulse, at 15 min after the stimulation started, led to EGF-QD and EEA1 co-localization level comparable to the control value (M1 ≈ 0.4), but then increased and, after 30 min, reached a value of about 0.5. Thereafter, the levels underwent a gradual decline, approaching the peak value observed in the control (Figure 8B). The BafA1 addition 15 min after the initiation of endocytosis resulted in an increase in co-localization by 10–15%, with only a marginal excess over the control. The BafA1 addition at subsequent phases exhibited a virtually negligible statistical impact. Activation of fusions during moderate deacidification of early endosomes (with the addition of BafA1 after 5–15 min) is also confirmed by the increased concentrating of EGF-QDs (Figure 8C). That is to say, an increase in pH to some intermediate value provokes fusions, which supports the assumption that the EEA1-positive early endosomes of enMSCs have a more acidic pH compared to HeLa. The association of EGF-QDs with the CD63 compartment of enMSCs also slowed down, and this effect is maximal upon addition of BafA15 min after the start of endocytosis, but it is largely similar to that under pretreatment conditions (Figure 4B and Figure 8D, dotted line). Addition of the inhibitor at later times makes the difference with the control curve even smaller (Figure 8D, yellow and orange curves, Supplementary Figure S6). It is important to emphasize that manipulations with the pH level in enMSC endosomes, although increasing the proportion of endocytic vesicles fusing with the EEA1-positive early endosomes, and thus entering the canonical pathway of lysosomal degradation, do not affect at least half of the population of newly formed vesicles carrying EGF-receptor complexes.

## 3. Discussion

MSCs are of significant interest for cell therapy because of their ability to self-renew and differentiate, as well as to secrete cytokines and signaling hormones, in particular, with angiogenic and anti-apoptotic effects [51]. It is widely accepted that the main signaling pathways regulating MSCs are Wnt- and TGF-*β*-dependent; however, the available evidence collectively indicates that EGFR and its ligands play a key role in regulating MSC proliferation and differentiation, as well as in enhancing their paracrine action [8,52,53,54,55]. Moreover, several reports suggest a role for EGFR in the regulation of the Wnt/*β*-catenin signaling pathway [56,57]. The majority of studies in this field have focused on the physiological outcomes of exogenously added EGF-like family ligands to cultured MSCs. These outcomes include the expression levels of some ligands and EGFR-family receptors (c-ErbB1-4, also known as HER1-4), their activation, and their stimulation/suppression of proliferation or differentiation [58,59,60]. However, the regulation of internalization and subsequent intracellular fate of EGF-receptor complexes is not practically considered in MSCs, although it can modulate cellular responses, both by decreasing the number of receptors on the surface membrane and by stimulating signals directly from endosomes. The paucity of data regarding endocytosis in MSCs in the existing literature renders the present study particularly relevant.

The basic concepts of endocytosis regulation and the role of endosomal acidification have been derived from the analysis of immortalized cells obtained from tumors, including HeLa, A431, and A549. The prevailing opinion is that the regulation of the endocytic apparatus, as well as of signaling in different cell types, is generally universal. However, our recent findings have revealed that EGF stimulates the internalization of ligand–receptor complexes in several types of MSCs, particularly those of endometrial origin, as well as in HeLa cells. Notably, the receptor degradation, which is BafA1-sensitive in both cases, took more than 6–8 h in MSCs, whereas in HeLa it took only 2–4 h [8].

Taking into account the importance of endolysosomal pH for several stages of the lysosomal degradative pathway, the present study focused on a comparative analysis of the effect of deacidification of the endolysosomal system by the specific V-ATPase inhibitor BafA1 on the endocytic fate of EGFR in tumor-derived (HeLa and A549) and undifferentiated enMSC cell lines. As a model of MSCs, we chose two lines of stromal mesenchymal cells from desquamated endometrium (enMSC2804 and enMSC2503) and the MSCs from endometrial biopsy (ECL2455) to be able to find some common features and their variations. The endocytosis of the EGFR was stimulated by adding the ligand to the control cells and to the cells with an already blocked V-ATPase under conditions of an increased pH level in the endolysosomal system. EGF labeled with QDs (EGF-QDs) in complex with the receptor was effectively internalized both in tumor-derived and enMSC cells, and deacidification did not inhibited this process. A detailed analysis of the dynamics of co-localization of EGF-receptor complexes with sequential compartments of the endocytic pathway was conducted using marker proteins that are typical for these compartments.

Two tumor-derived cell lines, HeLa and A549, which are widely used in endocytosis studies, demonstrated canonical type of EGF-receptor endocytosis with fast delivery of the bulk internalized EGF-QDs into EEA1-positive early endosomes, albeit with some variability in the dynamics (Figure 3), which may be explained with a particular Rab5 isoform, which functions predominantly in a cell line [61]. Our findings demonstrate that BafA1 addition, resulting in more basic endosomal pH, not only slowed down fusions of endocytic vesicles with early endosomes, but also the subsequent exit from them. It is evident that, consequently, the subsequent stages are shifted to a later time, but their order was shown to be not disturbed, and the delivery of cargo to lysosomes was not completely prevented. Therefore, the effect of BafA1 on cargo degradation is mostly due to the inhibition of lysosomal hydrolases activity, rather than the inhibition of cargo delivery to lysosomes.

In enMSCs, although a small proportion of newly formed endocytic vesicles enter the degradation pathway and exhibit basically the same patterns of sequential association with markers analogous to those observed in tumor-derived cells, key differences exist. Firstly, the higher number of endosomes formed after stimulation of endocytosis, their lower size/intensity and a very weak tendency to perinuclear clustering in enMSCs indicates that, under control conditions, the bulk of predominantly peripherally localized population of EGF-receptor-containing small endosomes do not undergo fusions early in the endocytic pathway. Secondly, stimulation of endocytosis in enMSCs pretreated with BafA1 does not reduce, but rather increases the association of EGF-QDs with EEA1 and “stabilizes” it, which leads to a slowdown in subsequent stages. More than that, our data suggests that, in proliferating enMSCs, there is a significant population of endosomes which is pH-independent, and this differs from the considered canonical EGFR endocytosis. It is important to note that this behavior is not a feature of endometrial MSCs; we observed a similar pattern in MSCs from dental pulp and Wharton’s jelly. Thirdly, although the level of EGF-QD association with CD63 in the control was higher than with other markers in all cell lines, the localization and morphology of CD63-positive structures differed in two cell types. In HeLa cells, CD63 was detected in the perinuclear region in EGF-QD-containing structures with CD63 and Lamp1 domains. EGF-QDs were redistributed with time, often together with CD63, to Lamp1-positive domains. In contrast with this, in enMSCs, CD63 was detected in both individual peripheral EGF-containing endosomes and vesicular structures localized around the nucleus (Figure 3 and Figure 4). In these cells, CD63-positive structures occupy a significantly larger area compared to Lamp1 vesicles, which indicates the presence of a population of CD63 vesicles in enMSCs that do not carry Lamp1. Furthermore, no accumulation of QDs was observed in Lamp1-positive lysosomes; as a result, BafA1 has virtually no effect on this parameter. These data suggest that, in enMSCs, fusions of newly formed endocytic vesicles with EEA1-positive early endosomes, as well as the transition from late endosomes which are positive for Rab7 and CD63, to Lamp1 lysosomes is ineffective and occurs at a slow rate.

Previously, it was believed that CD63 could be used as a lysosome marker due to its co-localization with Lamp1 in the perinuclear region [62,63,64]. However, the prevailing concept at present is that two CD63-positive subcompartments exist [65], one of which functions at the level of endo-/exocytosis and participates in the formation of exosomes and the selection of their cargo. The other contains a protein synthesized in the Golgi apparatus and is localized in fact in lysosomes. The relationship between these two subcompartments remains largely unknown; however, Artavanis-Tsakonas et al. reported a requirement for reduced pH in late phagosomes for recruitment of CD63 protein and its downregulation by BafA1 [66]. The inability to detect the co-localization of EGF-QDs with CD63 in the early peripheral endosomes of HeLa cells may be explained by the low concentration of tetraspanin.

The unexpected effect of early endosome deacidification on EGF-QD association with EEA1 in enMSCs suggests that their pH might normally be below the optimal pH for fusion, which is considered to be in the range of 6.2–6.8. As a result, endocytic vesicles in these cells after BafA1 pretreatment are exposed to conditions where the pH of early endosomes is within this range, thereby enhancing fusion efficiency. According to other authors, BafA1 does not lead to complete normalization of pH; however, its effects can vary depending on the number of proton pumps on the vesicle membrane [67], as well as on the isoform of protein *a*, a component of the membrane cluster of this ATPase, which acts as a pH sensor. Despite the discrepancies in the data, it has been demonstrated that, in early endosomes, the pump contains *a*_1_ [68] or *a*_2_ [23], while in late endosomes and lysosomes, it contains *a*_3_ [23,68], resulting in varying ATPase capacities.

In order to assess the hypothesis that the pH level in early endosomes of enMSCs is lower than in HeLa cells and does not provide an optimal range for fusion, we used a delayed addition of BafA1 (Figure 7A). Indeed, in enMSC2804, BafA1 addition immediately after the pulse resulted in an increase in the efficiency of ligand co-localization with EEA1, accompanied by an increase in cargo concentration per endosome (Figure 8). These data suggest that an increase in the pH to moderate value does increase the probability of early endosome fusions, thus supporting the idea of lower pH in early endosomes of enMSCs compared to tumor-derived cells (Figure 7 and Figure 8). However, this factor in enMSCs does not appear to be decisive for the delivery of the entire population of EGF-receptor complexes to EEA1-positive early endosomes and, respectively, directing them to the lysosomal degradation pathway. It can be suggested that the additive BafA1-dependent effect is provided at the expense of those EGF-QD-endosomes that initially associate with Rab5 but are unable to fuse with acidic EEA1-positive early endosomes under normal conditions. However, triple-co-localization experiments indicate that a fraction of endosomes that do not enter the EEA1-cycle remains present, even under more basic pH. The nature of this population requires further investigation; however, based on our preliminary data, this population possesses active receptor tyrosine kinase and can maintain prolonged EGFR signaling. However, our recent study showed that EGFR is finally degraded in a pH-dependent manner [8], and it is important to identify the ways that result in lysosomal delivery of the entire EGFR population in enMSCs. Obviously, further analysis of endocytic dynamics at longer times than were used in this study, as well as the investigation of known molecular regulators of late stages, are necessary.

Another important question concerns the specificity of late endosomal compartments’ organization. If in HeLa cells the dynamics of co-localization indicate a consequent transition from Rab7-vesicles to multidomain CD63/Lamp1 structures, followed by redistribution of EGF-QDs into Lamp1 territories; in enMSCs, the transition from Rab7 to CD63 appears less effective. This is even more true for CD63 to Lamp1 transitions. Potentially, vesicular rather than cluster organization of these late compartments results in less effective fusions. In HeLa cells under the action of BafA1, the formation of domains with triple co-localization is slowed down, possibly due to the suppression of Rab7 endosome redistribution to the perinuclear regions and/or the suppression of fusions of Rab7 and CD63 endosomes. In contrast to HeLa cells, control enMSCs exhibit a distinct population of EGF-QD/Rab7 vesicles that persists throughout the experiment, while single EGF-QD/CD63 endosomes are detected only at the earliest stage and are further found only in domains with triple co-localization. Deacidification significantly inhibits the formation of all three subpopulations (EGF-QDs/Rab7, EGF-QDs/CD63, and EGF-QDs/Rab7/CD63), but the most important difference is that upon BafA1 pretreatment; these populations of small vesicles are preserved along, with a few large structures exhibiting domain organization. Therefore, we hypothesize that deacidification in enMSCs negatively affects fusions at the late stage of endocytosis, which makes similar assumptions likely for HeLa cells.

Our data raises the question of the mechanisms that allow for EGF-receptor complexes to bypass endolysosome deacidification. The current understanding of the regulation of endocytosis by endosomal lumen acidification posits that effective fusions require calcium release from the endosome during the first minutes after internalization, and this process depends on V-ATPase activity and is suppressed by BafA1 [69]. Indeed, there is evidence of the presence of several Ca^2+^-permeable channels in endosomes (TPC1 and TPC3) and lysosomes (TPC2) [70]. Additionally, TRPV2 may function as an endosomal calcium release channel, thereby regulating endosome fusion [71,72]. Despite the small size of endosomes and lysosomes, which complicates experimental studies, it has become evident that their membranes carry a whole spectrum of ion channels and transporters, and the ion homeostasis of endolysosomes is regulated by the balance between the fluxes of K^+^, Na^+^, Cl^−^, Ca^2+^. Since the activity of V-ATPase is electrogenic, the cell must be able to maintain the electroneutrality of endolysosomes by exchanging protons for other cations. Accordingly, in the review by Mindel [73], the data were collected, allowing us to assume that, at relatively low acidification, this role is performed by the Na^+^/H^+^ exchanger (NHE), and, at high acidification, by the Cl^−^/H^+^ exchanger (CLC). It is also necessary to take into account the role of charged Donnan particles, represented by proteins localized in endosomes. Under suppression of the V-ATPase activity, they can provide a certain number of protons, ensuring incomplete deacidification of the endolysosome lumen, where all pH-dependent processes can be realized, albeit with reduced efficiency. All these assumptions obviously require extensive detailed studies to ascertain the mechanisms by which cells regulate endolysosome acidification, a process that is critical for their vital activities. It is also noteworthy that, according to certain data, BafA1 practically neutralizes acidification in early endosomes, but in late endosomes/lysosomes, the pH level increases only up to 6.0–6.5 [43,74,75,76]. These observations suggest that BafA1 exhibits distinct efficiencies on V-ATPase depending on the variant of the *a* subunit, and that there exist mechanisms for maintaining a reduced endolysosomal pH that are distinct from the activity of V-ATPase.

## 4. Materials and Methods

### 4.1. Cell Culture

Several human mesenchymal stem/stromal cell (MSC) lines were used. Two endometrial MSC lines enMSC2804 and enMSC2503 (tissue of origin is desquamated endometrium from menstrual blood) were obtained from MSC collection of the Department of Intracellular Signaling and Transport of the Institute of Cytology RAS, St. Petersburg, Russia. The ECL2455 mesenchymal stromal cells (tissue of origin is an endometrial biopsy) from the shared research facility, “Vertebrate cell culture collection” (Institute of Cytology RAS, St. Petersburg). These lines were previously obtained, and were shown to demonstrate properties typical of the MSC cultures [77,78]. The cells were cultured in DMEM/F12 medium (Gibco, Waltham, MA, USA) supplemented with 10% fetal bovine serum (FBS, Gibco, Waltham, MA, USA), 1% L-glutamine, and 1% penicillin–streptomycin (Gibco, Waltham, MA, USA) in an atmosphere of 5% CO_2_ at 37 °C. Cells from passages three to twelve were reseeded twice a week at a split ratio 1:3. These cells are characterized by a high proliferation rate (doubling time: 22–23 h).

HeLa cells (human cervix epidermoid carcinoma) and A549 cells (human epithelial lung carcinoma) from the “Vertebrate cell culture collection” (Institute of Cytology RAS, St. Petersburg, Russia) were maintained in DMEM (Gibco, Waltham, MA, USA) supplemented with 10% FBS (Gibco, Waltham, MA, USA) and 1% penicillin–streptomycin (Gibco, Waltham, MA, USA) in an atmosphere of 5% CO_2_ at 37 °C.

The cells were harvested by trypsinization for the experiments and plated on either Petri dishes with glass coverslips (Nunc, Thermo Scientific, Rochester, NY, USA) or 35 mm imaging dishes with a coverslip bottoms (Jet Biofil, Guangzhou, China), all in culture medium supplemented with 10% FBS. Experiments were performed at 60–70% confluence, 48 h after seeding.

### 4.2. Stimulation of Endocytosis

The EGF-biotin conjugate (EGF) and the CdSe/ZnS QD-streptavidin conjugate (QD) with an emission maximum at 655 nm were purchased from Invitrogen (Eugene, OR, USA). The EGF-QD complexes were prepared in vitro in PBS by mixing of EGF and QDs together for 30 min at 4 °C. The complexes were prepared using 8 nM EGF and 2 nM QDs.

The pulse-chase protocol was chosen to stimulate endocytosis under physiological conditions. The cells were washed twice with warm (37 °C) DMEM and pulsed for 5 min with EGF-QDs at 37 °C. The unbound ligands were then washed out thoroughly with warm DMEM, after which the cells were incubated for the indicated time at 37 °C before fixation (the total incubation time after stimulation is indicated).

### 4.3. Cell Treatments

Bafilomycin A1 (BafA1, Sigma-Aldrich, St. Louis, MO, USA), a specific inhibitor of vesicular proton pump V-ATPase, was added to the cells at a concentration of 100 nM for 30 min prior to endocytosis stimulation. For delayed addition, BafA1 was added to the cells 5, 15, or 30 min after endocytosis stimulation. In experiments with vital dyes, BafA1 was added to the cells for the specified periods of time. In all cases, after addition, BafA1 was present in the medium throughout the entire experiment.

### 4.4. Acidified Compartment Staining

To reveal late endosomes and lysosomes in living cells, the fluorescent vital dye acridine orange (AO, Merck, Darmstadt, Germany) was used at a concentration of 0.25 μg/mL. The cells were stained at room temperature for one min prior to confocal imaging.

In addition to AO, LysoTracker Red or LysoSensor Green DND-189 (Invitrogen, Eugene, OR, USA) at a concentration of 50 nM was used for vital staining of lysosomes and late endosomes. The cells were stained in an atmosphere of 5% CO_2_ at 37 °C for 20 min prior to confocal imaging.

### 4.5. Immunofluorescent Staining

The cells were fixed with 4% formaldehyde (Sigma-Aldrich, St. Louis, MO, USA) for 15 min, permeabilized with 0.5% Triton X-100 (SERVA Electrophoresis GmbH, Heidelberg, Germany) for an additional 15 min, and blocked with 1% bovine serum albumin (BSA, Sigma-Aldrich, St. Louis, MO, USA) for 30 min at room temperature. To reveal the EGFR and phospho-EGFR localization, the fixed cells were incubated with a primary anti-EGFR or anti-pEGFR (pY1068) antibody (1:100, Cell signaling technology, Danvers, MA, USA) for 24 h at 4 °C, followed by a 1 h incubation with Alexa 488 goat anti-rabbit IgG (1:500, Molecular Probes, Eugene, OR, USA). For co-localization analysis, the fixed cells were incubated with the following antibodies of choice: mouse anti-EEA1 (one hour at room temperature, 1:200, BD Biosciences, Franklin Lakes, NJ, USA), mouse anti-CD63 (one hour at room temperature, 1:200, Biolegend, San Diego, CA, USA), rabbit anti-Rab5 (24 h at 4 °C, 1:100, Cell signaling technology, Danvers, MA, USA), rabbit anti-Rab7 (24 h at 4 °C, 1:100, Cell signaling technology, Danvers, MA, USA), and rabbit anti-Lamp1 (24 h at 4 °C, 1:100, Cell signaling technology, Danvers, MA, USA). Subsequently, the cells were subjected to an additional hour of incubation with the secondary antibodies Alexa 488 or 405 goat anti-mouse IgG in 1:200 dilutions (Molecular Probes, Eugene, OR, USA) or Alexa 488 goat anti-rabbit IgG in 1:200 dilutions (Molecular Probes, Eugene, OR, USA). In triple-label co-localization experiments, the fixed cells were stained sequentially with two antibodies of choice (Rab5/EEA1, EEA1/Lamp1, Rab7/CD63 and CD63/Lamp1) according to the aforementioned protocols. Following the immunostaining procedure, the cells were mounted in Fluorescent Mounting Medium (DakoCytomation, Glostrup, Denmark).

### 4.6. Confocal Microscopy

Cell imaging was performed using an Olympus FV3000 laser scanning confocal microscope (Olympus, Tokyo, Japan). Samples were observed with a 40×/1.42 oil immersion objective, obtaining images with a resolution of 1024 × 1024 pixels. Images were captured in one, two, or three spectral channels in sequential scan mode, with only one laser operating at a time to avoid spectral overlap, and in a channel of differential interference contrast in transmitted light (DIC). Z-series optical sections were taken at 0.5 μm intervals from the bottom to the top of the specimen (14–20 sections).

QD fluorescence was excited at 405 nm and registered in the 640–670 nm range. The AO fluorescence was excited using 488 nm and 561 nm lasers and detected within two spectral regions: 500–550 nm and 620–680 nm. LysoSensor Green DND-189 was excited at 488 nm and recorded in the 500–550 nm range. LysoTracker Red fluorescence was excited at 561 nm and in the 570–670 nm range. Alexa 488 was excited at 488 nm and recorded in the 500–550 nm range. Alexa 405 was excited at 405 nm and recorded in the 410–480 nm range.

### 4.7. Image and Statistical Analysis

All data were obtained from a minimum of three independent experiments. In each experiment, 5–10 fields containing 20–60 cells in total were imaged for each time point. The images were processed and analyzed using Fiji software 1.52v (National Institutes of Health, Bethesda, MD, USA). In confocal images, adjustments to brightness and contrast were made exclusively for the purpose of presentation. The most representative projections of Z-stack onto a single image obtained using the max-intensity method (Fiji software 1.52v) were selected for demonstration of fixed cells. The most representative single sections from a Z-series of typical cells were selected for the demonstration of living cells. For quantitative analysis, raw images were utilized.

The analysis of endosomes selected as the Region of Interest (ROI) by thresholding procedure in each cell was carried out using Fiji software 1.52v. The perinuclear index of endosomes, the number of endosomes, and the mean integral intensity of QD fluorescence per endosome were calculated using Fiji software 1.52v (menu command Analyze Particles). The perinuclear index, defined as the ratio of the entire cell area to the area occupied by the bulk of endosomes, calculated for several time points, was used as a measure of the effectiveness of translocation of endosomes toward the microtubule organizing center.

The quantitative co-localization analysis was performed using Fiji software 1.52v JACoP Plugin to determine Manders’ co-localization coefficient (M1), which is defined using thresholds as the sum of intensities of co-localized pixels from one channel divided by their integrated density. The thresholds were established based on a visually estimated value for each channel.

The calculation of triple co-localization was performed using the following method: The co-localization of EGF-QDs in pairs with each of two antibodies was highlighted using plugin Colocalization (Fiji software 1.52v). For each pair, an 8-bit image was generated, containing exclusively the co-localized points. Subsequently, the co-localization between these two images is highlighted using plugin Colocalization (Fiji software 1.52v). Afterwards, the total area of all EGF-QD-positive structures per cell, as well as the areas with triple and double co-localization, were calculated using Fiji software version 1.52v (menu command Analyze Particles).

The statistical data processing was performed using Microsoft Office Excel 2021 (Microsoft Corporation, Albuquerque, NM, USA). The graphs and the bar charts were built using Microsoft Office Excel 2021. All results were obtained from at least three independent experiments. The results are presented as the mean ± standard error of the mean (SEM).

## 5. Conclusions

The data presented here indicate that the organization of the endolysosomal apparatus in canonical models for studying endocytosis of EGFR, such as immortalized HeLa cells, differs from that of undifferentiated proliferating enMSC cells from endometrium. In HeLa cells, internalized EGF-receptor complexes enter EEA1-endosomes, followed by delivery to late CD63-endosomes and final degradation in Lamp1-lysosomes. In enMSCs, only a limited fraction of EGFR-loaded endocytic vesicles demonstrates the same behavior as in HeLa cells. The remaining population consists of small, discrete vesicles that demonstrate low fusion efficacy and persist within the cell over prolonged period. We have demonstrated that BafA1 does not inhibit EGFR endocytosis; rather, it slows down EEA1-dependent fusions and the subsequent passage along the degradative pathway in HeLa cells. In enMSCs, deacidification stabilizes EGFR association with early endosomes. This finding may indicate a lower basal pH level in early endosomes of enMSCs, which is suboptimal for fusion. Another important finding is that, in contrast to HeLa cells, enMSCs exhibit a significant delay in the transition of EGFR from CD63-late endosomes to Lamp1-lysosomes. This stage may be responsible for the very slow degradation of EGFR and specific signaling in undifferentiated normal MSCs compared to tumor-derived cells.

The present study identifies the key stages of endocytosis at which the molecular causes of the observed differences should be sought. Since these stages are mostly dependent on intraluminal pH, both V-ATPase components and fusion regulation systems may be molecular targets of interest. In preliminary studies we have also observed a slowdown in EGFR degradation in other types of human MSCs. To understand how common this phenomenon is, it is necessary to expand the panel of MSCs of different tissue origins.

## Data Availability

The data presented in this study are available from the authors upon request.

## Supplementary Materials

The following supporting information can be downloaded at: https://www.mdpi.com/article/10.3390/ijms262010226/s1.

## Abbreviations

The following abbreviations are used in this manuscript:

BafA1	Bafilomycin A1
EGF	Epidermal Growth Factor
EGFR	Epidermal Growth Factor Receptor
enMSCs	Endometrial Mesenchymal stem/stromal cells
MSCs	Mesenchymal stem/stromal cells
MVBs	Multivesicular bodies
QDs	Quantum dots
