# Supplementary material for: Deacidification of the Endolysosomal System by the Vesicular Proton Pump V-ATPase Inhibitor Bafilomycin A1 Affects EGF Receptor Endocytosis Differently in Endometrial MSC and HeLa Cells
Source: Int J Mol Sci. 2025 Oct 21;26(20):10226. doi: 10.3390/ijms262010226 (PMC12563051; doi:10.3390/ijms262010226)
Supplement: Supplementary file 1 [file ijms-26-10226-s001.zip › ijms-3873496-supplementary.pdf]

Supplementary Materials: Deacidification of the Endolysosomal System by the Vesicular Proton Pump V-ATPase Inhibitor Bafilomycin A1 Affects EGF Receptor Endocytosis Differently in Endometrial MSC and HeLa Cells

Anna V. Salova [1,*], Tatiana N. Belyaeva [1], Ilia K. Litvinov [1], Marianna V. Kharchenko [1] and Elena S. Kornilova [1,2,*]

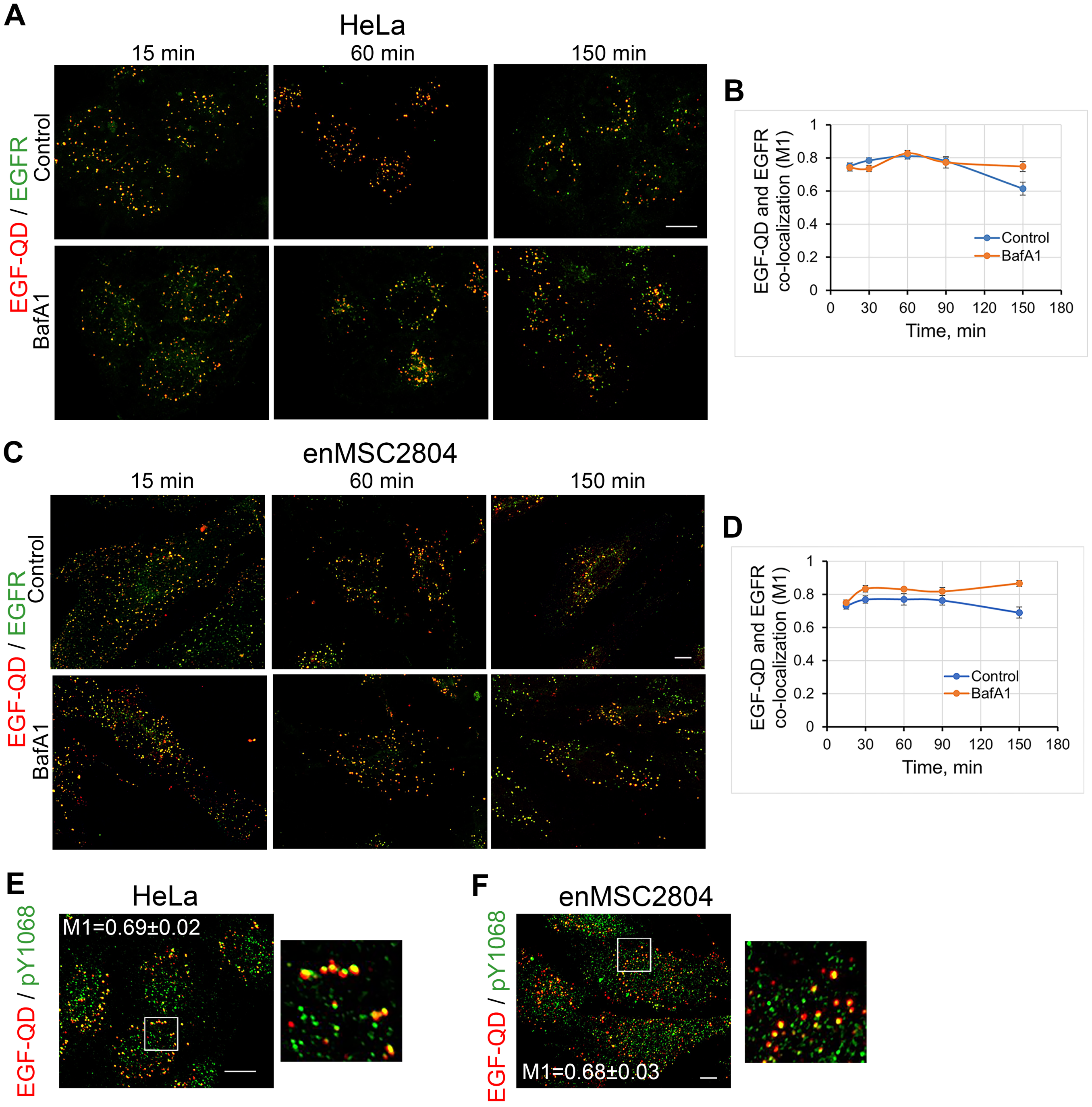

Figure S1. Analysis of the time-dependent distribution of EGFR after stimulation of endocytosis with EGF-QDs in HeLa and enMSC2804 cells in control conditions and after BafA1 pretreatment (100 nM, 30 min). (**A**, **C**) Confocal images of HeLa (**A**) and enMSC2804 (**C**) cells that were incubated with EGF-QDs (red) according to the experimental design described in Figure 2**A**. The cells were then fixed and immunostained with an anti-EGFR antibody (green). Each image is a maximum intensity projection of a Z-stack onto a single image, representative of the corresponding time point in at least three independent experiments. The brightness and contrast were only adjusted for presentation. Scale bars: 10 μm. (**B**, **D**) Manders' coefficients (M1) of co-localization between EGF-QDs and EGFR in HeLa cells (**B**, for a series of experiments with representative images in **A**) and enMSC2804 cells (**D**, for a series of experiments with representative images in **C**). All measurements were performed in three independent experiments, with a total of $\sim$ 100 cells per time point. The results are presented as the mean ± SEM. (**E**, **F**) Representative confocal images of HeLa (**E**) and enMSC2804 (**F**) cells after 30 min of endocytosis stimulation with EGF-QDs (red) according to the experimental design described in Figure 2**A**. The cells were fixed and immunostained with an anti-pEGFR (pY1068) antibody (green). Each image is a maximum intensity projection of a Z-stack onto a single image. The brightness and contrast were only adjusted for presentation. The enlarged (3.6$\times$) views of the corresponding boxed regions of the cell are presented. Scale bars: 10 μm.

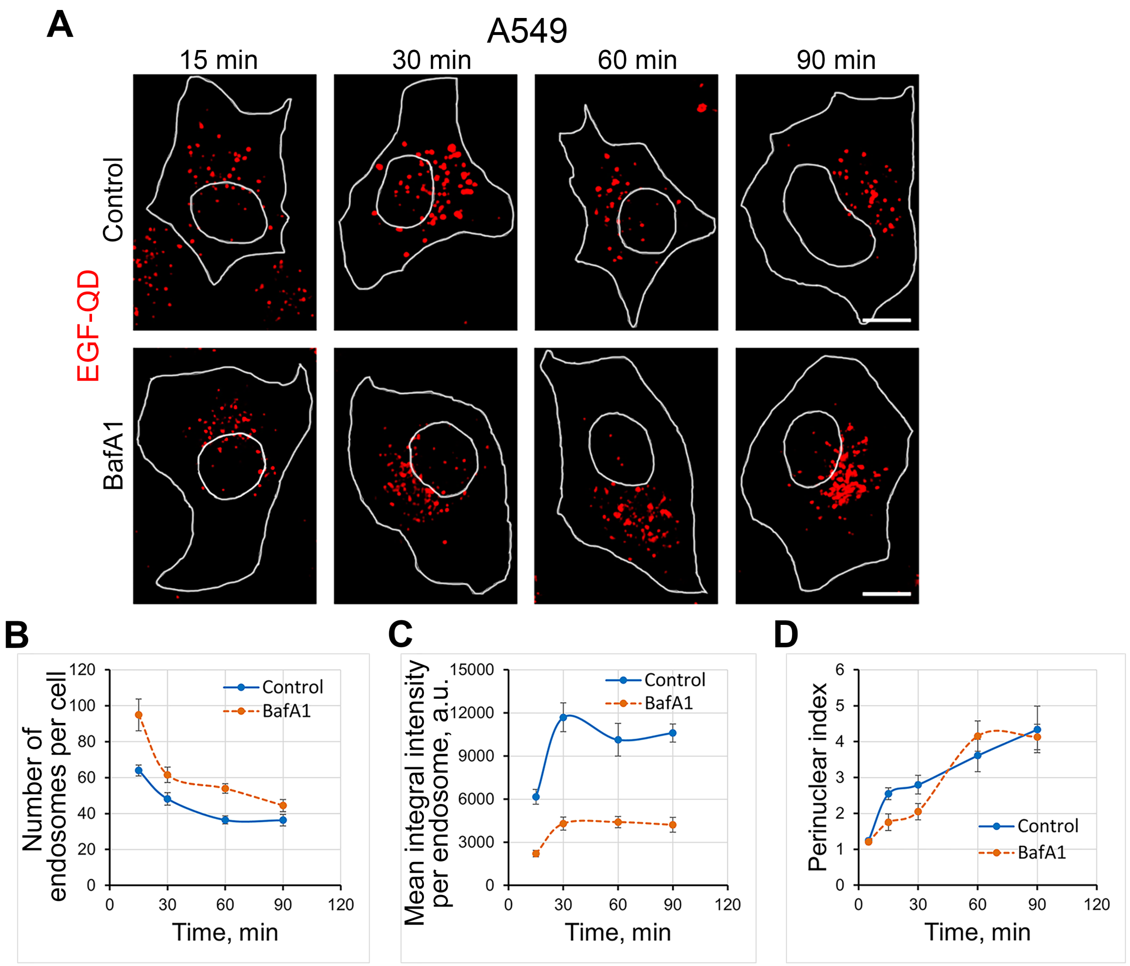

Figure S2. Time-dependent comparison of EGF-QD distribution during endocytosis in A549 cells in control conditions and after BafA1 pretreatment. (**A**) Confocal images of A549 cells that were incubated with EGF-QDs according to the experimental design described in Figure 2**A**. Each image is a maximum intensity projection of a Z-stack onto a single image, representative of the corresponding time point in at least three independent experiments. The brightness and contrast were only adjusted for presentation. The white lines in the images delineate the boundaries of the cells and nuclei according to differential interference contrast. Scale bars: 10 μm. (**B**) The number of endosomes per cell, (**C**) the mean integral intensity of QD fluorescence per endosome, and (**D**) the perinuclear index were calculated at each time point using Fiji software 1.52v for a series of experiments with representative images in **A**. All measurements were performed in three independent experiments, with a total of ∼ 150 cells per time point. The results are presented as the mean ± SEM.

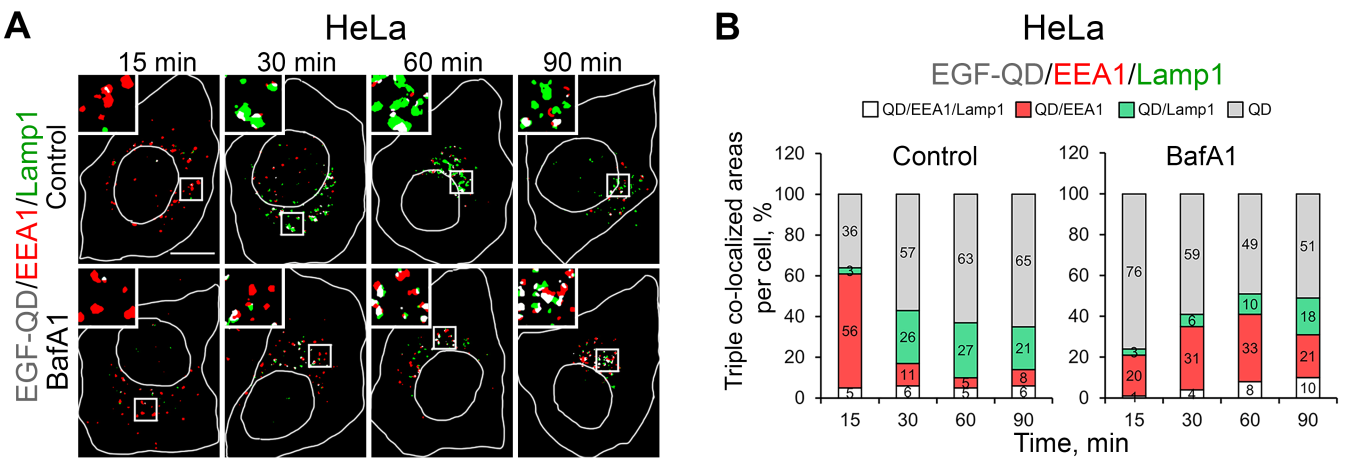

Figure S3. Triple co-localization analysis of the distribution of EGF-QDs in relation to specific endocytic markers EEA1 and Lamp1 in HeLa cells in control conditions and after BafA1 pretreatment (100 nM, 30 min). (**A**) Cell masks are shown after applying the triple co-localization detection method, revealing areas of co-localization of: QDs and EEA1 (red), QDs and Lamp1 (green), and the triple QDs, EEA1, and Lamp1 (white). The white lines in the images delineate the boundaries of the cells and nuclei according to differential interference contrast. The insets represent enlarged (3×) views of the corresponding boxed regions of the cell. Note that on the images only triple and double co-localized areas are presented. Scale bars: 10 μm. (**B**) Histograms showing the areas occupied by the QD-associated marker proteins EEA1 and Lamp1 per cell (triple and double co-localizations), normalized to the total QD-positive area, which include triple and double co-localization and vesicular structures containing only QDs, at each time point and presented as percentages in control conditions and after BafA1 pretreatment. All measurements were performed in three independent experiments, with a total of ∼ 100 cells per time point.

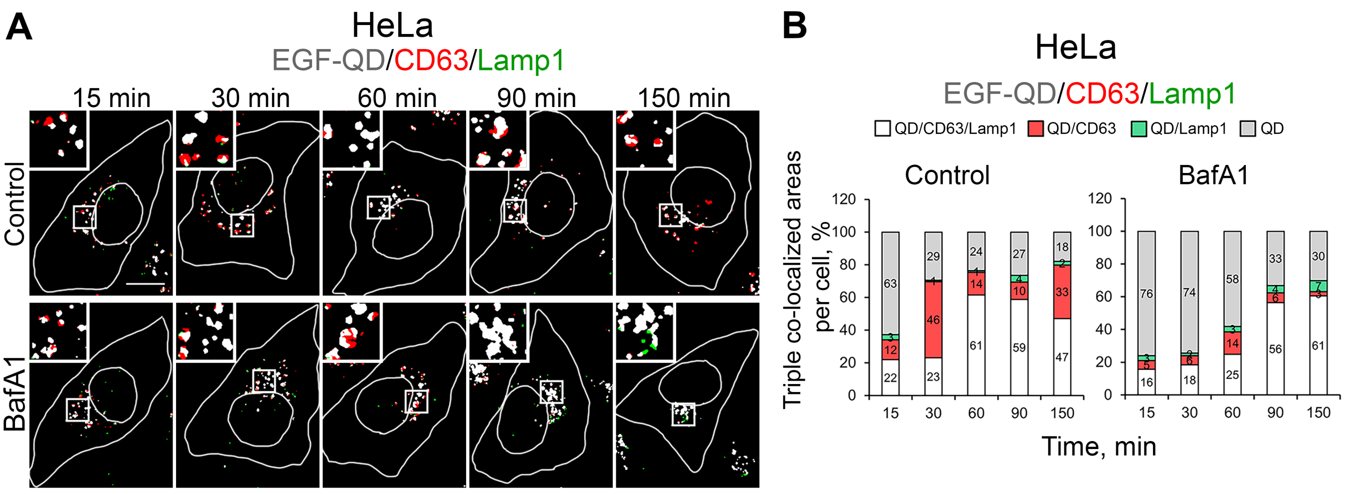

Figure S4. Triple co-localization analysis of the distribution of EGF-QDs in relation to specific endocytic markers CD63 and Lamp1 in HeLa cells in control conditions and after BafA1 pretreatment (100 nM, 30 min). (**A**) Cell masks are shown after applying the triple co-localization detection method, revealing areas of co-localization of: QDs and CD63 (red), QDs and Lamp1 (green), and the triple QDs, CD63, and Lamp1 (white). The white lines in the images delineate the boundaries of the cells and nuclei according to differential interference contrast. The insets represent enlarged (3×) views of the corresponding boxed regions of the cell. Note that on the images only triple and double co-localized areas are presented. Scale bars: 10 µm. (**B**) Histograms showing the areas occupied by the QD-associated marker proteins CD63 and Lamp1 per cell (triple and double co-localizations), normalized to the total QD-positive area, which include triple and double co-localization and vesicular structures containing only QDs, at each time point and presented as percentages in control conditions and after BafA1 pretreatment. All measurements were performed in three independent experiments, with a total of ∼ 100 cells per time point.

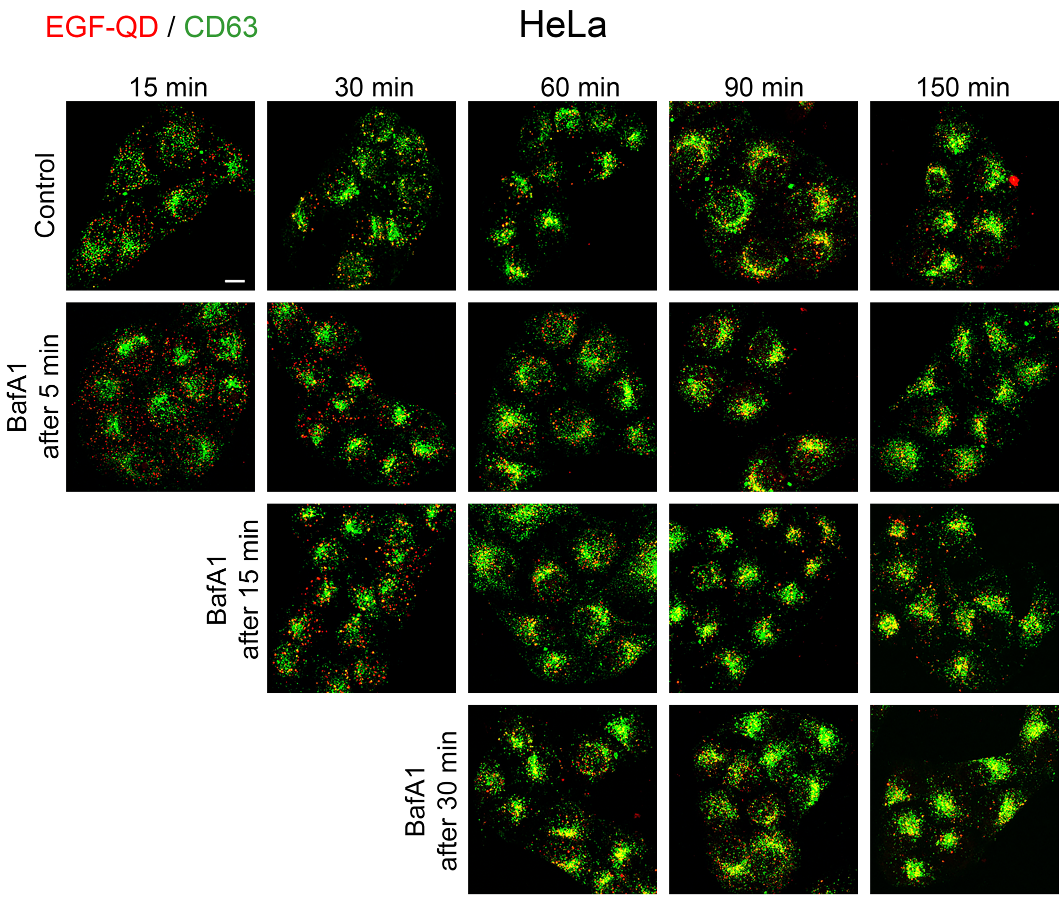

Figure S5. Delayed addition of BafA1 (100 nM) to HeLa cells after endocytosis stimulation with EGF-QDs. Confocal images of HeLa cells that were incubated with EGF-QDs (red) according to the experimental design for the delayed addition of BafA1 described in Figure 7**A**. The cells were then fixed and immunostained with an anti-CD63 antibody (green). Each image is a maximum intensity projection of a Z-stack onto a single image, representative of the corresponding time point in at least three independent experiments. The brightness and contrast were only adjusted for presentation. Scale bars: 10 µm.

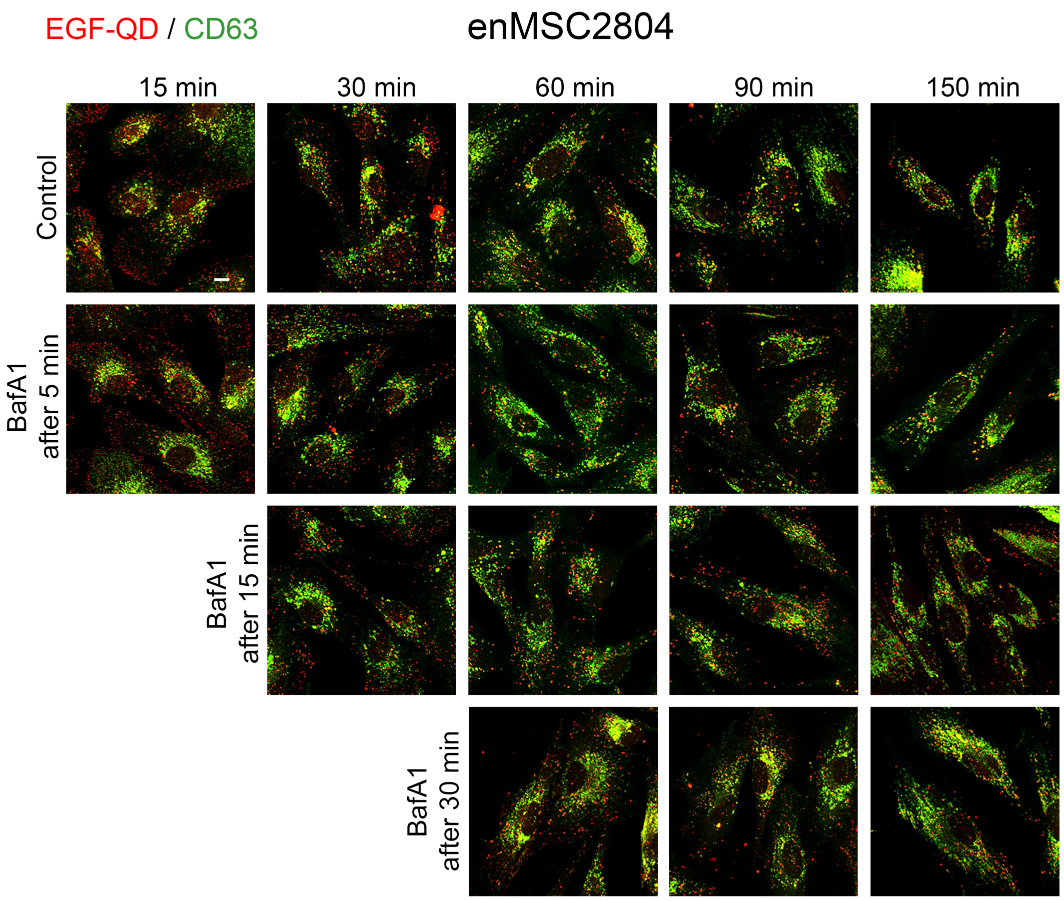

Figure S6. Delayed addition of BafA1 (100 nM) to enMSC2804 cells after endocytosis stimulation with EGF-QDs. Confocal images of enMSC2804 cells that were incubated with EGF-QDs (red) according to the experimental design for the delayed addition of BafA1 described in Figure 7**A**. The cells were then fixed and immunostained with an anti-CD63 antibody (green). Each image is a maximum intensity projection of a Z-stack onto a single image, representative of the corresponding time point in at least three independent experiments. The brightness and contrast were only adjusted for presentation. Scale bars: 10 µm.
